# Integration of Transcriptomic Analysis, Network Pharmacology, and Experimental Validation Demonstrates Enhanced Muscle-Protective Effects of Ethanol Extract of Jakyak-Gamcho-Tang

**DOI:** 10.3390/antiox14070795

**Published:** 2025-06-27

**Authors:** Aeyung Kim, Minh Nhat Tran, A Yeong Lee, Heerim Yeo, Su-Jin Baek, No Soo Kim, Seongwon Cha, Sang-Min Park

**Affiliations:** 1 KM Application Center, Korea Institute of Oriental Medicine, Daegu 41062, Republic of Korea; 2 College of Pharmacy, Chungnam National University, Daejeon 34134, Republic of Korea; tnminh@huemed-univ.edu.vn (M.N.T.); yeooh1@naver.com (H.Y.); 3 Faculty of Traditional Medicine, Hue University of Medicine and Pharmacy, Hue University, Hue 530000, Vietnam; 4 KM Data Division, Korea Institute of Oriental Medicine, Daejeon 34054, Republic of Korea; lay7709@kiom.re.kr (A.Y.L.); baeksj@kiom.re.kr (S.-J.B.); scha@kiom.re.kr (S.C.); 5 KM Convergence Research Division, Korea Institute of Oriental Medicine, Daejeon 34054, Republic of Korea; nosookim@kiom.re.kr

**Keywords:** Jakyak-gamcho-tang, muscle atrophy, transcriptome analysis, network pharmacology, oxidative stress

## Abstract

Muscle atrophy, characterized by progressive loss of skeletal muscle mass and strength, remains a significant therapeutic challenge. Jakyak-gamcho-tang (JGT) is a traditional herbal formulation that has demonstrated promising muscle-protective effects; however, the key bioactive constituents and the influence of different extraction methods have not yet been fully elucidated. This study compared the muscle-protective effects of the ethanol and water extracts of JGT (JGT-E and JGT-W, respectively), while also identifying the principal bioactive compounds that contribute to the enhanced efficacy of JGT-E. An integrative methodological approach was adopted, incorporating transcriptomic profiling, network pharmacology analysis, antioxidant activity assays, and in vitro validation using C2C12 myoblasts and myotubes. This comprehensive investigation enabled a detailed assessment of the biological activities of both JGT-E and JGT-W. Transcriptomic analysis revealed that JGT-E significantly modulates key pathways involved in oxidative phosphorylation, mitochondrial biogenesis, and signaling cascades related to PGC-1α, mTORC1, and ERRα, while simultaneously inhibiting TGF-β-mediated muscle atrophic signaling. Functional assays demonstrated that under oxidative stress conditions, JGT-E preserved mitochondrial content more effectively, reduced reactive oxygen species levels, and enhanced both myoblast viability and myotube integrity. Network pharmacology analysis identified isoliquiritigenin, catechin, and glabridin as major bioactive compounds enriched in JGT-E, all of which play critical roles in mitigating oxidative stress and supporting mitochondrial function. These findings were further substantiated by antioxidant assays that confirmed the contribution of these compounds to the observed muscle-protective effects of JGT-E. Overall, JGT-E exhibited superior efficacy in preventing muscle atrophy compared to JGT-W, likely due to its enriched profile of potent bioactive constituents. These results highlight the critical role of extraction methods in herbal medicine research and support the potential of JGT-E as a promising candidate for the treatment of muscle atrophy.

## 1. Introduction

Muscle atrophy, defined as the progressive loss of skeletal muscle mass and function [1], represents a significant clinical challenge that is commonly associated with aging, chronic diseases, and prolonged periods of immobilization [2]. This decline in muscle strength not only compromises quality of life but also increases the risk of morbidity and mortality [3]. Despite ongoing advances in biomedical research, the development of effective and safe therapeutic strategies to prevent or reverse muscle atrophy remains elusive [2]. Recently, traditional herbal medicines have garnered increasing attention as potential therapeutic alternatives, owing to their multi-target mechanisms and favorable safety profiles [4,5]. One such formulation, Jakyak-gamcho-tang (JGT), is a traditional decoction composed of Paeoniae Radix and Glycyrrhizae Radix et Rhizoma that has historically been used to relieve muscle cramps and spasms [6]. Emerging in vivo evidence has further highlighted the therapeutic potential of JGT, demonstrating its efficacy in attenuating muscle atrophy. Transcriptomic analyses have provided mechanistic insights into these effects, revealing that JGT modulates key pathways related to oxidative stress, mitochondrial function, and protein degradation [7].

A significant limitation of the current literature is its predominant reliance on water-based extraction methods, reflecting the traditional preparation practices for herbal formulations such as JGT [8]. However, the choice of extraction solvent plays a critical role in shaping the chemical profile of herbal extracts and consequently their biological activity. Water primarily extracts highly polar constituents such as glycosides and polysaccharides, whereas ethanol can solubilize a wider array of phytochemicals, including moderately polar and lipophilic compounds such as flavonoids, aglycones, and terpenoids [9]. These compositional differences have been shown to influence therapeutic efficacy substantially [10,11,12]. For example, the ethanol extract of *Rumex acetosa* contains significantly higher levels of emodin, a lipophilic anthraquinone that confers superior gastroprotective effects compared to its water extract [13]. Similarly, transcriptomic analysis of Bupleuri Radix demonstrated that while the water extract facilitated wound healing through promotion of cell adhesion and extracellular matrix organization, the ethanol extract predominantly activated apoptotic and anti-proliferative pathways, resulting in distinct therapeutic outcomes [14]. Such findings underscore the value of transcriptome-driven evaluation of extraction protocols, as facilitated by resources like KORE-MAP v1.0, which systematically profiles gene expression changes induced by herbal prescriptions under different extraction conditions [15]. Recent studies have also revealed considerable variation in the chemical composition of JGT depending on the extraction solvent. The ethanol extract (JGT-E) has shown significantly greater efficacy than the water extract (JGT-W) in scavenging reactive oxygen species (ROS) and protecting C2C12 myoblasts from oxidative-stress-induced cell death [16]. However, the specific bioactive compounds responsible for these enhanced antioxidative and muscle-protective effects have yet to be clearly identified.

The advent of network pharmacology has provided a robust and integrative framework for elucidating the complex, multi-component interactions that characterize traditional herbal medicines. This approach combines systems biology with computational modeling to predict compound–target interactions across a range of biological pathways, thereby aligning with the multi-target and multi-effect paradigm intrinsic to herbal formulations [17,18]. Through this methodology, the functional roles of specific phytochemicals in modulating disease-associated targets and signaling cascades have been systematically characterized. In the context of JGT, network pharmacology analysis has identified naringenin as a key bioactive compound in the treatment of functional dyspepsia. Naringenin was found to regulate gastrointestinal motility by modulating factors related to gastrointestinal movement, markers of interstitial cells of Cajal, and Aquaporin-3 expression [19]. Furthermore, the potential efficacy of JGT in addressing sphincter of Oddi dysfunction has been linked to the actions of glycycoumarin, licoflavonol, echinatin, and homobutein. These compounds are predicted to influence therapeutic outcomes by targeting the B cell receptor signaling pathway, the complement cascade, and muscle-contraction pathways [20]. These predictive insights not only deepen our mechanistic understanding of JGT’s therapeutic actions but also serve as a scientific basis for the rational design of targeted experimental validations.

Given that water decoction represents the traditional clinical preparation of JGT and ethanol extraction is widely adopted in modern pharmacology to capture less polar but bioactive constituents, this study aimed to systematically compare the muscle-protective effects of JGT-W and JGT-E. This comparative approach was intended to bridge traditional usage with chemical optimization. Additionally, this study sought to identify the key active constituents that may account for the differential effects observed between the two extracts. To address these objectives, we employed a comprehensive, integrative approach encompassing transcriptomic analyses, network pharmacology predictions, antioxidant activity assays, and experimental validation using C2C12 muscle cell models. This multifaceted strategy was designed not only to validate the enhanced efficacy of JGT-E but also to elucidate the specific molecular mechanisms and active compounds underlying its improved muscle-protective properties.

## 2. Materials and Methods

### 2.1. Chemicals

Ascorbic acid (A4544), 2,2′-Azino-bis(3-ethylbenzothiazoline-6-sulfonic acid) diammonium salt (ABTS, A1888), potassium persulfate (#216224), crystal violet solution (V5265), dexamethasone (D4902), dimethyl sulfoxide (DMSO, D8418), 2,2-diphenyl-1-picrylhydrazyl (DPPH, D9132), hydrogen peroxide (H_2_O_2_) solution (#216763), and sodium dodecyl sulfate (SDS, L3771) were obtained from Sigma-Aldrich (St. Louis, MO, USA). Palmitic acid (CFN99716) was obtained from Wuhan ChemFaces Biochemical (Wuhan, China).

### 2.2. Herbal Materials

Dried *Paeonia lactiflora* Pall. (Paeoniae Radix) and *Glycyrrhiza uralensis* Fisch. ex DC. (Glycyrrhizae Radix et Rhizoma) were obtained from Kwangmyungdang Co. (Ulsan, Republic of Korea). The quality of these specimens complies with the standards established by the Korean Pharmacopoeia. Reference specimens have been authenticated and are preserved under voucher numbers #2–21–0001 and #2–21–0002, respectively, at the Korean Herbarium of Standard Herbal Resources, which is affiliated with the Herbal Medicine Resources Research Center at the Korea Institute of Oriental Medicine (Naju, Republic of Korea).

### 2.3. Preparation of JGT-W and JGT-E

JGT-W and JGT-E were prepared by KOC Biotech Co. (Daejeon, Republic of Korea). In brief, JGT is composed of dried Paeoniae Radix and Glycyrrhizae Radix et Rhizoma in a 1:1 weight ratio. JGT-W was extracted with 10 times the volume of hot distilled water for 3 h using a reflux extraction system (MS-DM609; MTOPS, Seoul, Republic of Korea). JGT-E was produced by pulverizing the same materials, followed by the addition of 70% ethanol at a volume 4 times that of the sample weight. Ultrasonic extraction was conducted at 22–24 °C for 1 h, with this process being repeated twice. Both extracts were then filtered through a 53 µm mesh filter, concentrated using a rotary evaporator (Ev-1020, SciLab, Seoul, Republic of Korea) at 37 °C, and lyophilized in a freeze dryer (LP-20, Ilshin-Bio-Base, Dongducheon, Republic of Korea). The final extract was homogenized with a 600 µm sieve and stored in a foil Ziplock bag with desiccant silica gel. For the in vitro study, JGT-W and JGT-E (100 mg each) were vortexed for 30 min in 10 mL of phosphate-buffered saline (PBS; Thermo Fisher Scientific, Waltham, MA, USA) containing 2% DMSO, then sterilized through a 0.22 µm membrane. The resulting solution was aliquoted and stored at −80 °C until further use.

### 2.4. C2C12 Cell Culture and Differentiation

The C2C12 murine myoblast cell line (CRL-1772), originally derived from the thigh muscle of C3H mice after a crush injury, was obtained from the American Type Culture Collection (ATCC, Manassas, VA, USA). This cell line serves as a widely accepted in vitro model for studying skeletal muscle differentiation, oxidative stress, and muscle atrophy. C2C12 myoblasts were maintained in growth medium (GM) composed of high glucose (4.5 g/L) Dulbecco’s Modified Eagle Medium (DMEM) supplemented with 10% heat-inactivated fetal bovine serum and 100 IU/mL penicillin/100 μg/mL streptomycin (P/S) at 37 °C in a humidified 5% CO_2_ incubator. Upon reaching full confluence, cells were induced to differentiate into myotubes by switching to differentiation medium (DM) containing DMEM with 2% heat-inactivated horse serum and P/S. The DM was refreshed every 2 days for 5–7 days. All culture reagents were purchased from Thermo Fisher Scientific. 

### 2.5. Assessment of Cell Viability and Myotube Degradation in C2C12 Cells

To determine the optimal non-cytotoxic dose of JGT-W and JGT-E, 5000 cells of C2C12 myoblasts were plated into each well of a 96-well culture plate. After overnight incubation, the cells were treated with JGT-W and JGT-E at concentrations of 50, 100, 250, and 500 µg/mL, or with a vehicle control (0.1% DMSO). After 24 h, the supernatant was removed, and the cells were rinsed twice with PBS. Cell viability was determined using the EZ-Cytox Enhanced Cell Viability Assay Kit (Daeil Lab Service Co., Ltd., Seoul, Republic of Korea) and SpectraMax i3 microplate reader (Molecular Devices, LLC, Sunnyvale, CA, USA) following the manufacture’s guide. Based on these results and previous findings on muscle-protective activity of JGT-W [7], concentrations of 100 and 200 µg/mL were chosen for further experiments. To assess the protective role of JGT-W and JGT-E against oxidative stress-induced cellular damage, C2C12 myoblasts were pretreated with 100 and 200 µg/mL JGT-W and JGT-E for 12 h before being exposed to 0.25 mM H_2_O_2_. After 24 h, cell viability was assessed using EZ-Cytox Enhanced Cell Viability Assay Kit, and the results were expressed as a percentage relative to the vehicle-treated control cells. To investigate the protective effects of JGT-W and JGT-E on myotube degradation, C2C12 myotubes that had undergone differentiation for five days were pretreated with 100 and 200 µg/mL JGT-W and JGT-E for 12 h, followed by exposure to 0.25 mM H_2_O_2_, 0.2 mM dexamethasone, or 0.2 mM palmitic acid for an additional 24 h. After treatment, the cells were washed with PBS and stained with a crystal violet solution (0.2% crystal violet in 20% methanol) for 30 min at 25 °C. The stained cells were then rinsed with tap water, air-dried, and photographed. To quantify myotube density, the stained cells were solubilized in a 1% SDS solution at 37 °C for 30 min, and absorbance was measured at 590 nm using a SpectraMax i3 microplate reader.

### 2.6. Transcriptomic Analysis

The RNA-seq data of C2C12 myotubes treated with increasing concentrations of JGT-W and JGT-E (20, 100, and 500 μg/mL) for 24 h were obtained from GEO GSE227494 and GSE295069, respectively. Bioinformatics analysis of the RNA-seq data was performed using the R (v4.2.1). Differential gene expression analysis between groups was conducted using the DESeq2 package (v1.38.2), resulting in a ranked list of genes based on fold change and adjusted *p*-value corrected for multiple testing via false discovery rate. Gene set enrichment analysis determines the degree to which genes within a defined gene set that share a common biological function or pathway are disproportionately overrepresented at the top or bottom of a specific ranked list of genes, resulting in a normalized enrichment score (NES) and a false discovery rate (FDR). The gene sets of Hallmark, Gene Ontology, and canonical pathways were obtained from the Molecular Signature Database (MSigDB). Gene set enrichment analysis was performed using the fgsea package (v1.24.0) with the following parameters: 10,000 permutations, minimum size 15, and maximum size 500. GSEA results were visualized by the heatmap package (v.1.0.12).

### 2.7. Detection of Intracellular ROS Level

To evaluate the protective effects of JGT-W and JGT-E against oxidative stress, C2C12 myoblasts and myotubes were pretreated with 100 and 200 µg/mL of JGT-W and JGT-E for 12 h. Following this pretreatment, the cells were subjected to oxidative stress by exposure to 0.25 mM H_2_O_2_ for 6 h. The levels of intracellular ROS were measured using the CellROX^®^ Green Reagent (Thermo Fisher Scientific) in accordance with the manufacturer’s protocol. In brief, after exposure to H_2_O_2_, the cells were incubated with 5 µM CellROX^®^ Green for 30 min at 37 °C. Subsequently, the cells were washed with Hanks’ balanced salt solution. Fluorescence images were obtained using a fluorescence microscope (IX71, Olympus Co., Tokyo, Japan), and the fluorescence intensity was quantified using ImageJ software, version 1.54f (National Institute of Health, Bethesda, MD, USA).

### 2.8. Measurement of Mitochondrial Mass

C2C12 myotubes were pretreated with JGT-W and JGT-E for 12 h and then exposed to 0.25 mM H_2_O_2_ for 24 h. To evaluate mitochondrial mass, the cells were washed with PBS and then incubated with 50 nM MitoTracker™ Deep Red FM (M22426, Thermo Fisher Scientific) in serum-free DMEM for 30 min at 37 °C. After washing with PBS, red fluorescence images were captured using a fluorescence microscope, and the fluorescence intensity was quantified using ImageJ software, version 1.54f.

### 2.9. Free Radical Scavenging Assay

The free radical-scavenging activities of JGT-W and JGT-E were evaluated using ABTS and DPPH assays. For the ABTS assay, a stock solution was prepared by mixing equal volumes of ABTS (7.4 mM) and potassium persulfate (2.6 mM) in the dark for 2 h. The working solution was diluted to an optical density of ~0.7 at 734 nm (OD734). Serially diluted JGT-W and JGT-E were combined with the ABTS working solution in a 96-well plate and incubated in the dark for 7 min at 22–24 °C, after which OD734 was measured using a SpectraMax i3 microplate reader. In the DPPH assay, a working solution of 0.2 mM DPPH was prepared in absolute methanol in the dark. Equal volumes of serially diluted JGT-W and JGT-E were mixed with the DPPH solution and incubated for 30 min at 22–24 °C, followed by measurement at OD514. Ascorbic acid was used as a positive control. Free radical-scavenging activities were calculated using the formula: inhibition (%) = (1 − OD of JGT-W, JGT-E, or ascorbic acid/OD of distilled water) × 100.

### 2.10. Quantitative Analysis of JGT Using UHPLC-MS/MS

Quantitative analyses of 16 components of JGT were carried out using an Agilent 1290 Infinity II ultrahigh-performance liquid chromatography (UHPLC) system and 6495 C triple quadrupole mass spectrometer (Agilent Technologies, Santa Clara, CA, USA). The sample was detected as positive and negative modes of electrospray ionization (ESI) source. A UHPLC column was used to Kinetex 1.7 μm C18 100A (150 × 2.10 mm; Phenomenex, Torrance, CA, USA), set to 40 °C. The temperature of the auto-sampler was kept at 4 °C, and injection volume was 2 μL. Water with 0.1% formic acid (A) and acetonitrile (B) were used as mobile phase applying a gradient program; 5–20% B for 0–1 min, 20–25% B for 1–6.5 min, 25–50% B for 6.5–17 min, 50–100% B for 17–20 min, isocratic 100% B for 20–25 min, 100–5% B for 25–26 min, and isocratic 5% B for 26–30 min. The capillary voltage was set to positive of 3500 V and negative of 3000 V. Nozzle voltage of positive and negative was 500 V and 1500 V, respectively. Both gas temperature and nebulizer gas was set to 130 °C and 25 psi. Drying gas flow was 11.0 L/min, and the MS/MS parameters of 16 standard components were optimized using dynamic multiple reaction monitoring (MRM), shown in Table 1. The software used for quantitative analysis was MassHunter version 10.1. The 16 components for quantitative analysis were 1,2,3,4,6-O-Pentagalloylglucose (CFN90192), benzoylpaeoniflorin (CFN99536), glabridin (CFN99731), glycyrrhizin (CFN99151), isoliquiritin apioside (CFN90800), liquiritin apioside (CFN90707), ononin (CFN99136, ≥98.0%; ChemFaces, Hubei, China), albiflorin (HY-N0037, ≥98.67%); 18beta-glycyrrhetinic acid (HY-N0180, ≥99.88%); isoliquiritigenin (HY-N0102, ≥ 98.07%), Oxypaeoniflorin (HY-N0748, ≥98.37%; MedChemExpress, Princeton, NJ, USA), isoliquiritin (A0041), liquiritigenin (A0042), liquiritin (A0040, ≥99.0%; Chengdu Must Bio-Technology Co., Ltd., Chengdu, China), (+)-catechin (PHR1963, USP reference standard), and paeoniflorin (P0038, ≥98.0%; Merck KGaA, Darmstadt, Germany). All standards were mixed and quantitatively analyzed using a simultaneous multi-compound analysis method. To verify the analytical accuracy, recovery tests were conducted, and data are summarized in Supplementary Table S1.

### 2.11. Network Pharmacology Analysis

A network pharmacology approach was employed to investigate the potential compounds and mechanisms of action of JGT in relation to muscle atrophy. Information on 16 compounds identified from HPLC analysis was retrieved from PubChem, including compound names, IDs, and SMILES structures. The targets of these compounds were collected and predicted using TCMSP v2.3, HIT 2.0, BATMAN-TCM 2.0, and SwissTargetPrediction v2019. Experimentally validated known targets were extracted from HIT, TCMSP, and BATMAN-TCM, ensuring comprehensive coverage of bioactive interactions. Additionally, predicted targets from TCMSP were also collected, while in BATMAN-TCM, those with a score cutoff ≥ 0.84 were included. SwissTargetPrediction was utilized to predict targets based on SMILES structures, and only those with a probability > 0.5 were selected for further analysis. To identify potential therapeutic targets related to muscle atrophy, data were extracted from DisGeNET v24.4, GeneCards v5.23, TTD v2024, and CTD (2025 updated). Targets were selected based on specific cutoff criteria, including a gene-disease associations score > 0.1 in DisGeNET, a Relevance score > 10 in GeneCards, and an Inference Score > 60 in CTD, while all available targets from TTD were included. For pathway enrichment analysis, Gene Ontology Biological Process (GOBP) enrichment was performed using enrichGO in R, with significantly enriched pathways selected based on an adjusted *p*-value (*p* < 0.05, Benjamini–Hochberg correction). The top 10 terms were prioritized based on adjusted *p*-values, providing insight into the biological processes influenced by the identified targets. Finally, herb-compound–target networks were constructed and analyzed using Cytoscape v3.10.3 to visualize and explore the interactions among compounds and target proteins, enabling the identification of potential compounds involved in muscle atrophy-related mechanisms.

### 2.12. Statistical Analysis

Data analysis was conducted with GraphPad Prism (v 9.5.1, GraphPad Software, San Diego, CA, USA). The results are expressed as the mean ± standard deviation (SD). Group differences were assessed using one-way analysis of variance, followed by Dunnett’s multiple comparison test, with statistical significance established at *p* < 0.05.

## 3. Results

### 3.1. Transcriptomic Analysis Shows That JGT-E Regulated Muscle-Protective Pathways Under Oxidative Stress More Effectively than Jgt-W

**Figure 1:**
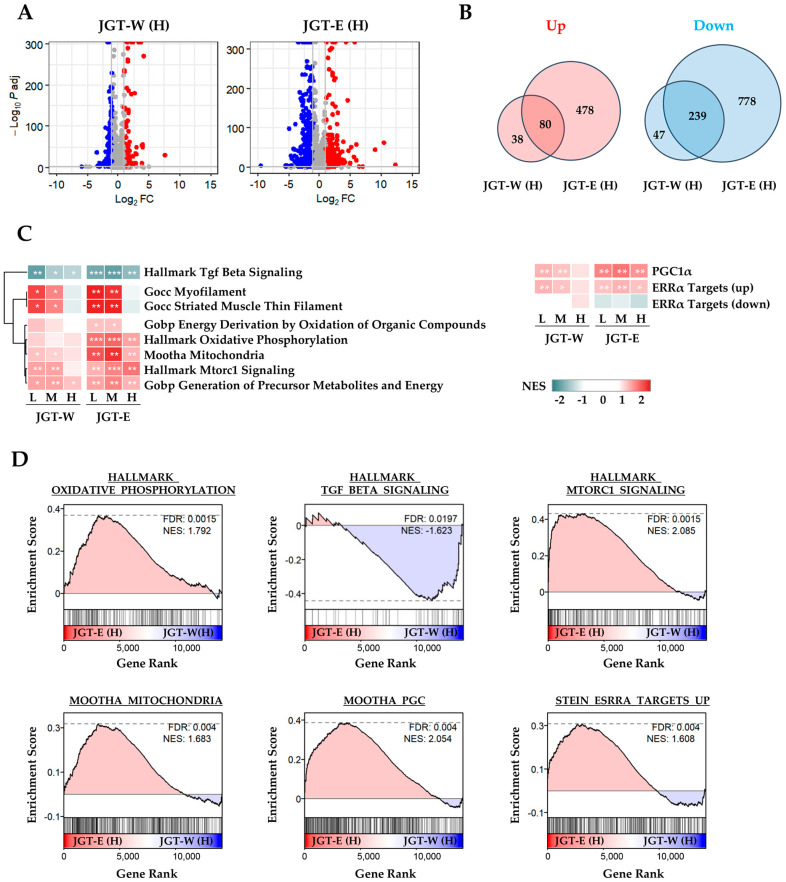
Transcriptomic analysis of C2C12 muscle cells treated with JGT-W or JGT-E. (**A**) Volcano plots depicting differentially expressed genes (DEGs) in C2C12 myotubes treated with JGT-W or JGT-E at a high dose (H). Genes significantly upregulated or downregulated (|log_2_ fold-change| > 1, adjusted *p* < 0.05) are marked in red or blue, respectively. (**B**) Venn diagrams illustrating the overlap and differences between significantly upregulated (left) and downregulated (right) DEGs from JGT-W and JGT-E treatments. (**C**) Gene set enrichment analysis (GSEA) results comparing enriched molecular pathways between JGT-W and JGT-E-treated cells at low (L, 20 μg/mL), medium (M, 100 μg/mL), and high (H, 500 μg/mL) doses. Normalized enrichment score (NES) is indicated by color intensity. Statistical significance of the false discovery rate (FDR) is indicated by asterisks: * FDR < 0.05, ** FDR < 0.01, *** FDR < 0.001. (**D**) Representative GSEA plots highlighting key pathways, including oxidative phosphorylation, TGF-β signaling, mitochondrial function, PGC-1α signaling, mTORC1 signaling, and ERRα targets. The dotted horizontal line in each GSEA plot represents the maximum or minimum enrichment score for the gene set across the ranked gene list.

To compare the muscle-protective effects of JGT-W and JGT-E, we performed transcriptomic profiling of differentiated C2C12 myotubes. Differential gene expression analysis revealed that treatment with JGT-E resulted in the regulation of a significantly larger number of genes compared to JGT-W, indicating a stronger transcriptional response (Figure 1A). While both extracts shared several transcriptional signatures, JGT-E uniquely modulated a broader set of genes, implying a more extensive impact on gene expression (Figure 1B). Pathway enrichment analysis revealed clear dose-dependent enrichment of key molecular pathways implicated in muscle protection (Figure 1C). Notably, cells treated with JGT-E exhibited robust upregulation of pathways related to oxidative phosphorylation, mitochondrial function, PGC-1α signaling, mTORC1 signaling, and ERRα targets, along with marked downregulation of muscle-atrophic TGF-β signaling (Figure 1D). Furthermore, JGT-E significantly enhanced mitochondrial metabolic processes, cellular respiration, and ATP biosynthesis across all tested concentrations (Supplementary Figure S1A,B). Genes associated with mitochondrial biogenesis and myogenic differentiation were also more significantly changed by JGT-E (Supplementary Figure S1C). Collectively, these findings imply that the enriched bioactive components in JGT-E may be responsible for its superior muscle-protective efficacy compared to JGT-W.

### 3.2. JGT-E Demonstrated Greater Efficacy than JGT-W in Mitigating Cell Damage and ROS Production in C2C12 Myoblasts Under Oxidative Stress

**Figure 2:**
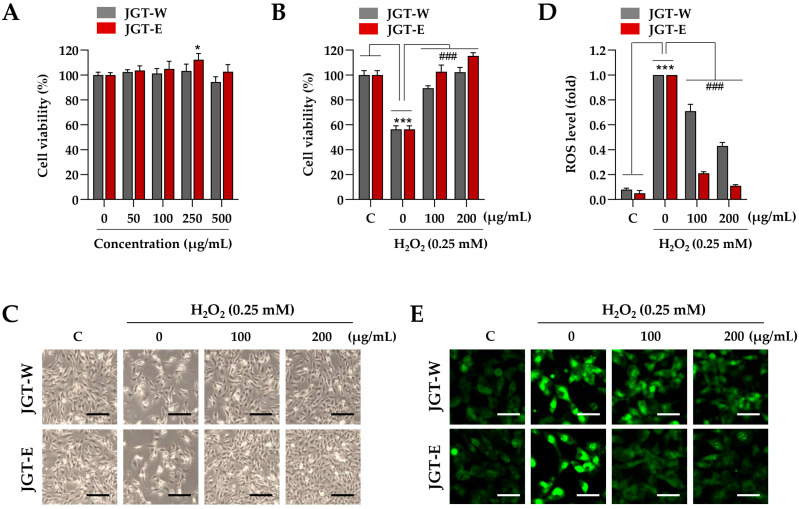
Effects of JGT-W and JGT-E under oxidative stress condition in C2C12 myoblasts. (**A**) C2C12 myoblasts were treated with increasing concentrations of JGT-W and JGT-E, ranging from 0 to 500 μg/mL. After 24 h, relative cell viability, compared to vehicle-treated control cells, was determined. (**B**,**C**) Myoblasts were pretreated with 100 or 200 μg/mL of JGT-W, JGT-E, or vehicle for 12 h, followed by exposure to 0.25 mM H_2_O_2_. After 24 h, relative cell viability was assessed. (**B**) Additionally, cellular morphology was captured using an inverted microscope (**C**). (**D**,**E**) C2C12 myoblasts were pretreated with 100 or 200 μg/mL of JGT-W, JGT-E, or vehicle for 12 h, and then exposed to 0.25 mM H_2_O_2_. After 6 h, intracellular levels of ROS were assessed using the ROS-sensitive CellROX^TM^ Green dye. The fold increase in ROS levels relative to the vehicle-treated controls was quantified using ImageJ, version 1.54f. (**D**) The production of intracellular ROS was visualized through green fluorescence under a fluorescence microscope (**E**). All data are presented as the mean ± SD (*n* = 3). Statistical significance was evaluated by one-way ANOVA followed by Dunnett’s multiple comparison test. * *p* < 0.05, *** *p* < 0.001 vs. vehicle-treated control cells; ### *p* < 0.001 vs. H_2_O_2_ + vehicle-treated control cells. Scale bar = 100 μm.

Building on transcriptomic evidence demonstrating superior muscle-protective effects of JGT-E over JGT-W, we further validated these findings using a C2C12 muscle-cell model. To determine the appropriate treatment concentration, C2C12 myoblasts were incubated with various concentrations of JGT-W and JGT-E, up to 500 µg/mL, for 24 h. Cell viability assays indicated no cytotoxicity at concentrations up to 250 µg/mL (Figure 2A), and thus, 200 µg/mL was selected as the maximum concentration for subsequent in vitro experiments. To evaluate the protective effects of JGT-W and JGT-E under oxidative stress, C2C12 myoblasts were pretreated with either extract or a vehicle control (0.1% DMSO) for 12 h, followed by exposure to 0.25 mM H_2_O_2_ for 24 h. As shown in Figure 2B, H_2_O_2_ treatment significantly reduced cell viability to approximately 56.3% of control levels. In contrast, pretreatment with JGT-W and JGT-E markedly mitigated cytotoxic damage. At concentrations of 100 µg/mL and 200 µg/mL, JGT-W preserved cell viability at approximately 89.4% and 102.3% of control levels, while JGT-E maintained it at 102.6% and 115.4%, respectively. Morphological analysis further confirmed these protective effects: cells pretreated with either JGT-W or JGT-E exhibited minimal morphological changes following H_2_O_2_ exposure, closely resembling the morphology of untreated control cells (Figure 2C). Intracellular ROS levels were assessed using CellRox™ Green dye. As expected, H_2_O_2_ exposure induced marked green fluorescence, indicative of elevated ROS levels. Pretreatment with both extracts significantly attenuated ROS accumulation in a dose-dependent manner, with JGT-E demonstrating superior efficacy (Figure 2D). Specifically, at 100 µg/mL, JGT-W reduced ROS levels by approximately 29%, while JGT-E achieved a 79% reduction. At 200 µg/mL, the reductions were approximately 57% for JGT-W and 89% for JGT-E (Figure 2E). Collectively, these findings indicate that both JGT-W and JGT-E confer protective effects against oxidative stress-induced cytotoxicity in myoblasts, with JGT-E exhibiting significantly greater efficacy.

### 3.3. JGT-E Was More Effective than JGE-W in Promoting Mitochondrial Biogenesis, Reducing ROS Levels, and Inhibiting the Degradation of C2C12 Myotubes Exposed to H_2_O_2_

**Figure 3:**
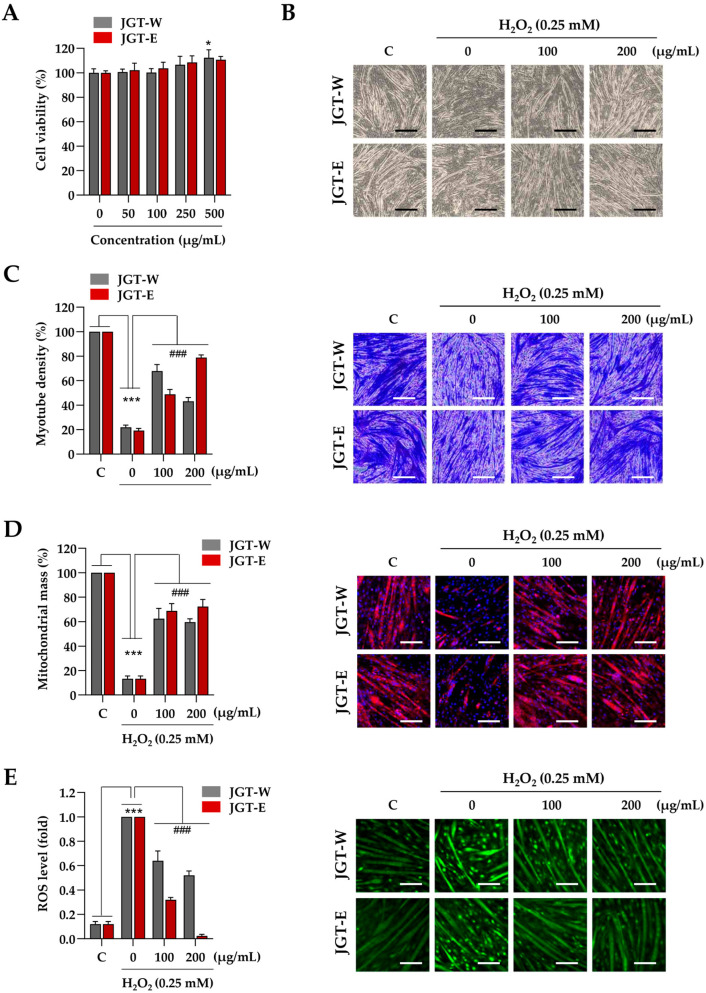
Effects of JGT-W and JGT-E under oxidative stress condition in C2C12 myotubes. (**A**) C2C12 myotubes were treated with specified concentrations of JGT-W and JGT-E. After 24 h, the cell viability, relative to the vehicle-treated control cells, was determined. (**B**–**D**) C2C12 myotubes were pretreated with 100 and 200 μg/mL of JGT-W and JGT-E for 12 h and subsequently exposed to 0.25 mM H_2_O_2_ for an additional 40 h. Morphological changes were observed using an inverted microscope (**B**). Myotube density was quantified through crystal violet staining. (**C**) After staining with MitoTracker^TM^ Deep Red, mitochondrial mass was visualized under a fluorescence microscope and quantified using ImageJ, version 1,54f. (**D**,**E**) C2C12 myotubes pretreated with 100 and 200 μg/mL of JGT-W and JGT-E for 12 h were subsequently exposed to 0.25 mM H_2_O_2_. After 6 h, intracellular ROS levels were detected using CellROX^TM^ Green dye under a fluorescence microscope. The fold increase in ROS levels was quantified using Image J, version 1,54f. All data are presented as the mean ± SD (*n* = 3). Statistical significance was evaluated by one-way ANOVA followed by Dunnett’s multiple comparison test. * *p* < 0.05, *** *p* < 0.001 vs. vehicle-treated controls; ### *p* < 0.001 vs. H_2_O_2_ + vehicle-treated controls. Scale bar = 100 μm.

Next, C2C12 myotubes were treated with JGT-W and JGT-E at concentrations of up to 500 µg/mL for 24 h, during which time no cytotoxic effects were observed (Figure 3A). Accordingly, 200 µg/mL was selected as the maximum concentration for subsequent experiments, consistent with the protocol used in C2C12 myoblast studies. To assess the protective effects of JGT-W and JGT-E against oxidative-stress-induced muscle atrophy, myotubes were pretreated with 100 µg/mL or 200 µg/mL of either extract for 12 h, followed by exposure to 0.25 mM H_2_O_2_ for 24 h. As illustrated in Figure 3B, H_2_O_2_ treatment resulted in pronounced myotube degradation, characterized by morphological collapse and shortening. In contrast, pretreatment with JGT-W or JGT-E significantly ameliorated these effects and preserved the characteristic morphology of the myotubes, as confirmed by crystal violet staining (Figure 3C). Quantitative analysis showed that myotube density decreased by approximately 79.1% following H_2_O_2_ exposure compared to vehicle-treated controls. Pretreatment with 100 µg/mL or 200 µg/mL of JGT-W restored myotube density to 67.8% and 43.2%, respectively. Notably, pretreatment with JGT-E at the same concentrations preserved myotube density at 48.9% and 78.9%, respectively, indicating a more robust protective effect. To investigate further the impact on mitochondrial integrity, mitochondrial mass was assessed using MitoTracker™. As shown in Figure 3D, H_2_O_2_ markedly diminished mitochondrial mass in C2C12 myotubes; however, pretreatment with either JGT-W or JGT-E substantially restored mitochondrial content even after oxidative insult. Additionally, pretreatment with JGT-W or JGT-E significantly reduced H_2_O_2_-induced intracellular ROS accumulation in a dose-dependent manner, with JGT-E demonstrating superior efficacy (Figure 3E). Under conditions of chemically induced muscle atrophy using dexamethasone and palmitic acid, both JGT extracts significantly attenuated myotube degradation and increased myotube density. In these models, JGT-E consistently exhibited slightly greater efficacy than JGT-W (Supplementary Figure S2A). Collectively, these results indicate that both JGT-W and JGT-E effectively mitigate oxidative stress-induced myotube damage, with JGT-E showing marginally superior muscle-protective effects. In addition, we validated our injury models by evaluating two established positive control compounds, resveratrol and arachidonic acid [21,22,23], under identical conditions. Pretreatment with either compound significantly inhibited myotube degradation caused by H_2_O_2_, dexamethasone, and palmitic acid. These results, shown in Supplementary Figure S2B, confirm the robustness of our in vitro atrophy models and establish a pharmacological benchmark for the muscle-protective potential of JGT extracts.

### 3.4. Quantitative Analysis of JGT-W and JGT-E Using UHPLC-MS/MS

**Figure 4:**
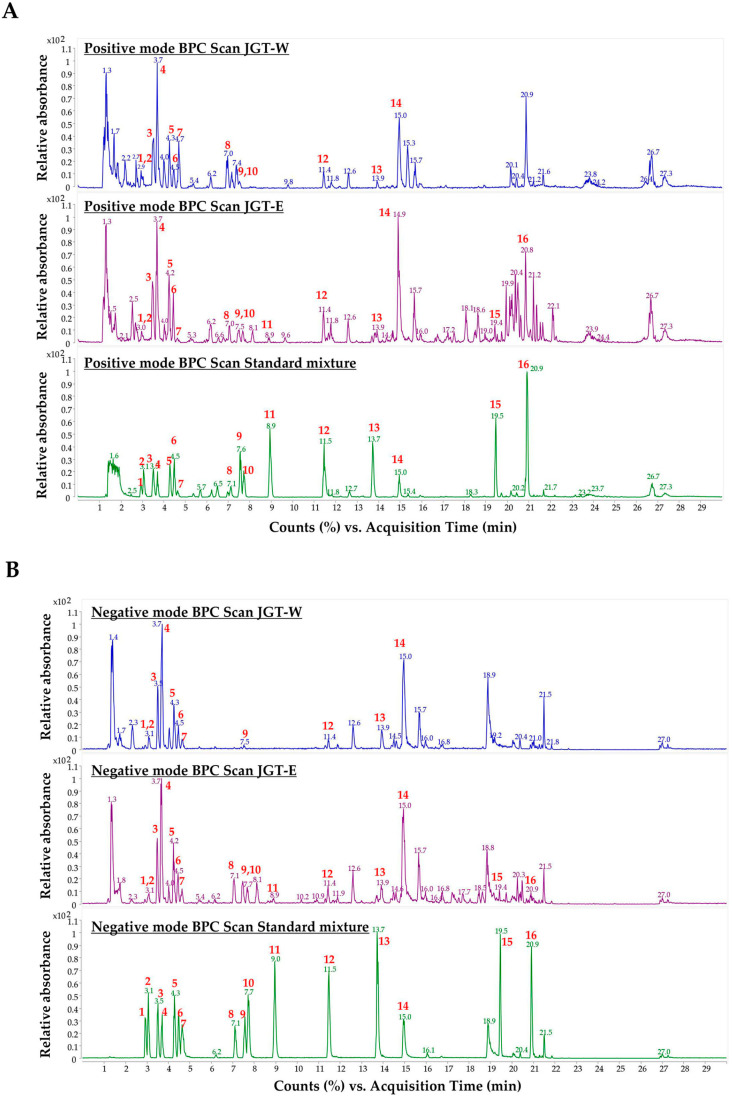
Analysis of JGT-W and JGT-E using UHPLC-MS/MS. Positive-ion mode of base peak ion chromatograms (BPC) (**A**) and negative-ion mode of BPC (**B**) of JGT-W, JGT-E, and standard mixture using a UHPLC-TQ-MS/MS. Red numbers indicate standard compounds; 1: oxypaeoniflorin, 2: (+)-catechin, 3: albiflorin, 4: paeoniflorin, 5: liquiritin apioside, 6: liquiritin, 7: 1,2,3,4,6-pentagalloyl glucose, 8: isoliquiritin apioside, 9: ononin, 10: isoliquiritin, 11: liquiritigenin, 12: benzoylpaeoniflorin, 13: isoliquiritigenin, 14: glycyrrhizin, 15: glabridin, 16: glycyrrhetinic acid.

To characterize the primary constituents of JGT-W and JGT-E, LC-MS/MS was conducted at a concentration of 1.00 mg/mL. Samples exceeding the calibration curve range were subjected to a 10-fold dilution. Quantitative analysis of 16 target compounds was performed using a dynamic MRM method coupled with a UHPLC-triple quadrupole mass spectrometer. Representative chromatograms of JGT-W, JGT-E, and the standard mixture are presented in Figure 4 and Figure S3, while the quantitative data for all 16 compounds are summarized in Table 2. In JGT-W, isoliquiritin, liquiritigenin, and glycyrrhetinic acid were detected at concentrations below the limit of quantification and were, therefore, reported as trace amounts. Overall, most compounds were present at higher concentrations in JGT-E than in JGT-W. Notably, albiflorin and oxypaeoniflorin were detected at slightly higher levels in JGT-W. Paeoniflorin was the most abundant compound in JGT-W, whereas glycyrrhizin was predominant in JGT-E.

### 3.5. Prediction of Potential Compounds in JGT Involved in Muscle-Protective Mechanisms

**Figure 5:**
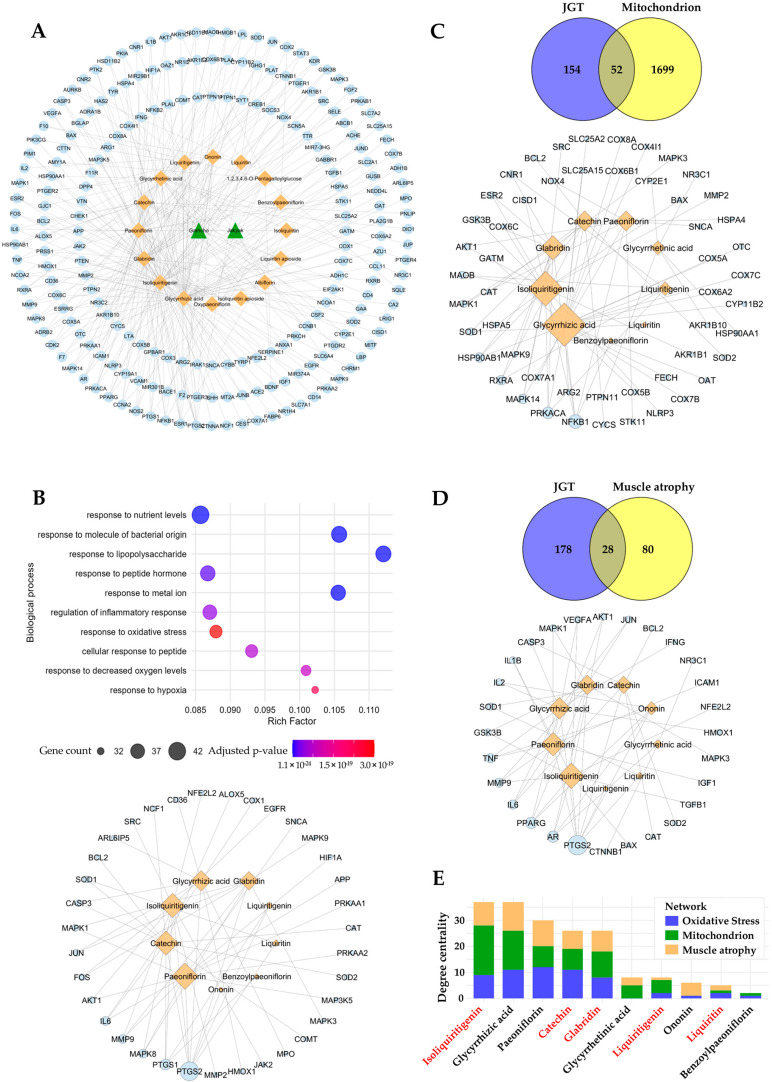
Network pharmacology-based prediction of compounds in JGT effective for muscle atrophy. (**A**) The herb-compound–target network of JGT, where green triangle nodes represent herbs (2 nodes), orange diamond nodes represent compounds (16 nodes), and light blue circle nodes represent targets (206 nodes). (**B**) Gene Ontology Biological Process (GOBP) enrichment analysis of the 206 JGT-associated targets (top) and the compound–target network (bottom), focusing on 35 targets linked to the “Response to Oxidative Stress” (GO:0006979) pathway. (**C**) Overlap between JGT targets and mitochondria-related genes (52 overlapping targets) (top), along with the compound–target network of JGT compounds interacting with these overlapping mitochondrial targets (bottom). (**D**) Overlap between JGT targets and muscle atrophy-related genes (28 overlapping targets) (top), with the corresponding compound–target network of JGT compounds interacting with these overlapping muscle atrophy targets (bottom). The node sizes represent the degree centrality of each node in networks (**B**–**D**). (**E**) Total degree centrality of JGT compounds across the three networks (**B**–**D**): oxidative stress, mitochondrion, and muscle atrophy. Compounds highlighted in red are present at higher concentrations in JGT-E compared to JGT-W.

To identify the bioactive compounds responsible for the enhanced muscle-protective efficacy of JGT-E, we performed a network pharmacology analysis. In total, 206 unique molecular targets were retrieved from 16 compounds in JGT, after eliminating duplicates across multiple databases (Figure 5A). Pathway enrichment analysis of these targets revealed significant associations with oxidative stress responses (GO:0006979, adjusted *p*-value = 3.04 × 10^−19^, gene count = 35), implying strong antioxidant potential for the constituents of JGT (Figure 5B, top). To identify the compounds involved in modulating this pathway, we constructed a compound–target interaction network focused on the 35 oxidative-stress-associated targets of JGT (Figure 5B, bottom). Among the top-ranking compounds (degree > 7) identified were paeoniflorin, isoliquiritigenin, catechin, glycyrrhizic acid, and glabridin. To investigate further whether these key compounds also regulate mitochondrial function and muscle-atrophy-related pathways, we expanded our analysis by constructing additional compound–target networks based on mitochondrial- and muscle-atrophy-related targets (Figure 5C,D). These analyses consistently highlighted the importance of the top bioactive compounds across multiple relevant pathways. To assess the significance of each compound comprehensively across the three biological contexts (oxidative stress, mitochondrial function, and muscle atrophy), we aggregated the degree centrality of each compound (Figure 5E). This analysis revealed that isoliquiritigenin, catechin, glabridin, and liquiritigenin—compounds that are more abundant in JGT-E—ranked among the most influential bioactive constituents, with isoliquiritigenin emerging as the most pivotal overall. Collectively, these network pharmacology findings imply that the superior muscle-protective effects of JGT-E, compared to JGT-W, are likely attributable to its higher concentrations of these key bioactive compounds, which notably enhance the modulation of oxidative-stress-, mitochondrial-function-, and muscle-atrophy-related pathways.

### 3.6. Identification of Compounds That Contribute to Protection Against Oxidative Stress and Muscle Damage

**Figure 6:**
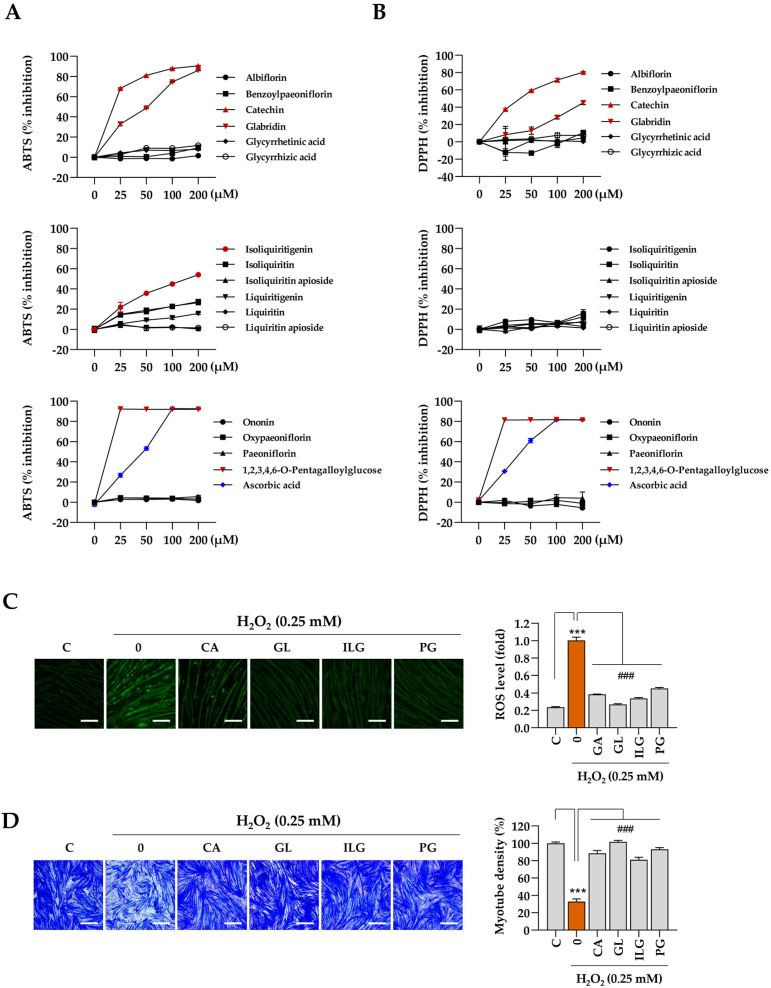
Antioxidant and muscle-protective activity of compounds in JGT. (**A**,**B**) Free radical-scavenging activities of 16 compounds were evaluated using the ABTS (**A**) and DPPH (**B**) assays. Ascorbic acid was used as a positive control in both assays. (**C**) C2C12 myotubes were pretreated with non-toxic concentrations of catechin (CA, 50 μM), glabridin (GL, 10 μM), isoliquiritigenin (ILG, 20 μM), or 1,2,3,4,6-pentagalloyl glucose (PG, 20 μM) for 12 h and subsequently exposed to 0.25 mM H_2_O_2_ for 6 h. Intracellular ROS were detected following labeling with CellROX^TM^ Green dye. (**D**) C2C12 myotubes pretreated with CA, GL, ILG, or PG were incubated for an additional 40 h in the presence of 0.25 mM H_2_O_2_. After staining with crystal violet solution, myotube density was quantified. Data are presented as the mean ± SD (*n* = 3 except for free radical scavenging assay, *n* = 2). Statistical significance was evaluated by one-way ANOVA followed by Dunnett’s multiple comparison test. *** *p* < 0.001 vs. vehicle-treated controls; ### *p* < 0.001 vs. H_2_O_2_ + vehicle-treated control cells. Scale bar = 100 μm.

The muscle-protective properties of the bioactive compounds identified in JGT were further evaluated based on the results from the network pharmacology analysis. Initially, cell-free biochemical assays were conducted to assess the antioxidant capacity of the 16 compounds, with a focus on their free-radical scavenging activity, using established methods such as the ABTS and DPPH assays. The results indicate that catechin, glabridin, isoliquiritigenin, and 1,2,3,4,6-pentagalloyl glucose exhibited significant efficacy in ABTS radical scavenging (Figure 6A), while catechin, glabridin, and 1,2,3,4,6-pentagalloyl glucose demonstrated notable effectiveness in DPPH free-radical scavenging (Figure 6B). Ascorbic acid, used as a positive control, showed dose-dependent free-radical scavenging activity in both assays. Subsequently, we assessed the ability of these four selected compounds (catechin, glabridin, isoliquiritigenin, and 1,2,3,4,6-pentagalloyl glucose) to modulate intracellular ROS production under oxidative stress induced by H_2_O_2_. The results show a significant reduction in intracellular ROS levels following pretreatment with these compounds (Figure 6C). To evaluate further the protective effects of the 16 compounds in JGT against H_2_O_2_-induced myotube degradation, C2C12 myotubes were pretreated with the compounds for 12 h, followed by exposure to 0.25 mM H_2_O_2_. After 24 h, the cells were stained with crystal violet, and myotube density was quantified (Supplementary Figure S4). As shown in Figure 6D, myotube density was maintained at approximately 88.4%, 101.6%, 81%, and 93% of control levels for catechin, glabridin, isoliquiritigenin, and 1,2,3,4,6-pentagalloyl glucose, respectively. These findings demonstrate that these compounds effectively preserve muscle tissue, which may contribute to the superior muscle-protective efficacy of JGT-E compared to JGT-W.

## 4. Discussion

JGT shows promise as a therapeutic option for muscle atrophy, as demonstrated by the efficacy of JGT-W in our previous research [7]. However, the specific roles of the individual active compounds within JGT remain insufficiently defined, and the impact of different extraction methods on its therapeutic properties has not been comprehensively explored. In this study, both transcriptomic and network pharmacology analyses consistently revealed that JGT-E exhibited superior muscle-protective effects compared to JGT-W. Transcriptomic analysis demonstrated that JGT-E regulated a greater number of genes than JGT-W, significantly enhancing pathways related to oxidative phosphorylation, mitochondrial function, PGC-1α, mTORC1, and ERRα signaling, while concurrently suppressing muscle-atrophic TGF-β pathways (Figure 1).

Our previous research demonstrated that JGT-W provides protection against dexamethasone-induced muscle atrophy by promoting muscle protein synthesis, inhibiting degradation, and preserving mitochondrial integrity and bioenergetic function [7]. In the current study, transcriptomic data indicate that JGT-E exerted even more pronounced effects on these protective mechanisms. Specifically, JGT-E upregulated genes associated with oxidative phosphorylation, mitochondrial biogenesis, PGC-1α signaling, mTORC1 signaling, and ERRα targets, while simultaneously downregulating muscle-atrophic TGF-β signaling (Figure 1D). Mitochondrial biogenesis, which is crucial in maintaining mitochondrial mass and function, directly impacts skeletal muscle health. Any impairment of this process can lead to muscle dysfunction. Mitochondrial dysfunction is regulated by complex signaling networks, including the AMPK-SIRT1-PGC-1α, IGF-1-PI3K-Akt-mTOR, FoxOs, JAK-STAT3, TGF-β-Smad2/3, and NF-κB pathways [24]. Among these, PGC-1α activation, a key regulator of mitochondrial biogenesis [25], supported the hypothesis that JGT-E would enhance mitochondrial maintenance and function. This finding aligns with previous studies showing that PGC-1α protects skeletal muscle from atrophy by inhibiting FoxO3 signaling. Moreover, PGC-1α activation has been shown to prevent denervation-induced muscle atrophy [26] and age-related sarcopenia [27]. Additionally, the activation of mTORC1 signaling in cells treated with JGT-E further implies its role in promoting muscle protein synthesis and anabolic growth. mTORC1 regulates protein and lipid synthesis, supporting cellular growth, and its decline during muscle disuse or immobilization results in reduced protein synthesis and subsequent atrophy [28,29]. TGF-β, a key regulator of fibrosis and inflammation, induces skeletal muscle atrophy and fibrosis by activating Atrogin-1 and Scleraxis [30]. Therefore, the suppression of TGF-β signaling by JGT-E may contribute to its superior muscle-protective effects. Collectively, these findings highlight JGT-E as a more potent therapeutic candidate than JGT-W, likely due to its enhanced regulation of mitochondrial function and anabolic signaling, and its inhibition of muscle-atrophic pathways. To validate the findings from the transcriptome analysis, we performed a comparative evaluation of the muscle-protective effects of JGT-W and JGT-E under conditions of oxidative stress-induced muscle damage, using a C2C12 mouse muscle-cell line model. Our results demonstrate that both JGT-W and JGT-E significantly reduced intracellular ROS production in C2C12 myoblasts and myotubes. This resulted in preserved cell viability in myoblasts, increased myotube density, and enhanced mitochondrial content in the myotubes. Importantly, JGT-E demonstrated superior muscle-protective effects compared to JGT-W (Figure 2 and Figure 3).

These transcriptomic changes were further supported by the differential expression of key genes associated with mitochondrial biogenesis and myogenic differentiation, as shown in Figure S1C. JGT-E treatment markedly upregulated Sirt1, Esrra, Mfn1, and Dnm1l, which play essential roles in mitochondrial function and remodeling. Notably, Sirt1 enhances the activity of PGC-1α through deacetylation, while Esrra (encoding ERRα) acts as a downstream effector of PGC-1α to promote oxidative metabolism [31,32]. These gene-level changes are consistent with the increased mitochondrial content observed in JGT-E-treated myotubes (Figure 3D). In addition, the elevated expression of Myog and Myod1, two master regulators of myogenic differentiation, corresponds to the preserved myotube morphology and increased density under oxidative stress (Figure 3C). The suppression of Foxo3, a pro-atrophic transcription factor [33], may further contribute to reduced expression of muscle-specific E3 ubiquitin ligases and lower ROS levels, as observed in both myoblasts (Figure 2E) and myotubes (Figure 3E). Taken together, these findings provide mechanistic insight into how the transcriptional reprogramming induced by JGT-E leads to improved mitochondrial integrity, reduced oxidative damage, and preservation of muscle fiber structure.

Network pharmacology analysis has identified several key compounds in JGT, including isoliquiritigenin, glycyrrhizic acid, paeoniflorin, catechin, and glabridin, which play significant roles in oxidative-stress responses, mitochondrial function, and regulation of muscle atrophy (Figure 5). Notably, isoliquiritigenin, catechin, and glabridin were found in significantly higher concentrations in JGT-E compared to JGT-W (Table 2). These compounds have potent anti-inflammatory and antioxidant properties, and their protective effects on muscle have been substantiated in both in vitro and in vivo studies [34,35,36,37,38]. Isoliquiritigenin exhibits potent antioxidant effects and improves mitochondrial function through activation of the Nrf2 signaling pathway in aging-related neurodegenerative diseases [39,40]. For musculoskeletal diseases, isoliquiritigenin has been shown to mitigate dexamethasone-induced muscle atrophy by increasing myotube diameters and decreasing expression of Atrogin-1 and MuRF-1 through the Akt/mTOR signaling pathway [41]. Catechin enhances skeletal muscle mass and strength by promoting mitochondrial biogenesis, which provides adequate energy for physiological functions and reduces the oxidative stress associated with aging, disease, and acute exercise [42]. Catechin can also enhance muscle regeneration by activating and promoting the differentiation of muscle stem cells [43], while also mitigating muscle atrophy by upregulating anabolic factors and increasing muscle fiber cross-sectional area [44]. Additionally, glabridin has been reported to inhibit dexamethasone-induced protein degradation in C2C12 myotubes and muscle atrophy in murine models. It does so by attenuating the expression of MuRF-1 and Cbl-b through suppression of glucocorticoid receptor activation. As an antagonist, glabridin binds directly to the glucocorticoid receptor, preventing its activation and subsequent downstream signaling [45]. These findings imply that the enhanced efficacy of JGT-E in the treatment of muscle atrophy may be primarily attributed to the elevated concentrations of these bioactive compounds, which collectively contribute to mitochondrial protection, reduction in oxidative stress, and preservation of muscle integrity.

The investigation into the free-radical scavenging activity of 16 compounds in JGT revealed that catechin, glabridin, and isoliquiritigenin exhibited significant antioxidant properties, which aligned with the primary compounds identified through network pharmacology analysis (Figure 6A,B). These compounds were shown to reduce ROS production and mitigate myotube degradation induced by H_2_O_2_ (Figure 6C,D). Additionally, 1,2,3,4,6-pentagalloyl glucose was selected for its notable antioxidant activity and its ability to protect against myotube degradation. Importantly, the contents of these four compounds were found to be higher in JGT-E than in JGT-W, which may contribute to the enhanced efficacy observed in JGT-E.

One limitation of network pharmacology analysis is its reliance on available data, which may result in underrepresentation of certain bioactive compounds with fewer known or predicted targets. As a consequence, some compounds that demonstrate promising effects in experimental studies may not be adequately represented in network analyses; 1,2,3,4,6-O-pentagalloylglucose is an example of such a compound. Therefore, additional research is needed to better understand its mechanisms and ensure a more comprehensive evaluation of its therapeutic potential. Another limitation of this study is the absence of in vivo validation. While the current study focused on in vitro analyses, our previous work has already confirmed the in vivo efficacy of JGT-W in a dexamethasone-induced muscle atrophy model [7]. Given the enhanced effects of JGT-E and its key bioactive constituents, such as isoliquiritigenin, catechin, and glabridin, demonstrated in this study, follow-up evaluations in diverse preclinical models of muscle injury are required to further verify their therapeutic potential and safety profiles.

## 5. Conclusions

**Figure 7:**
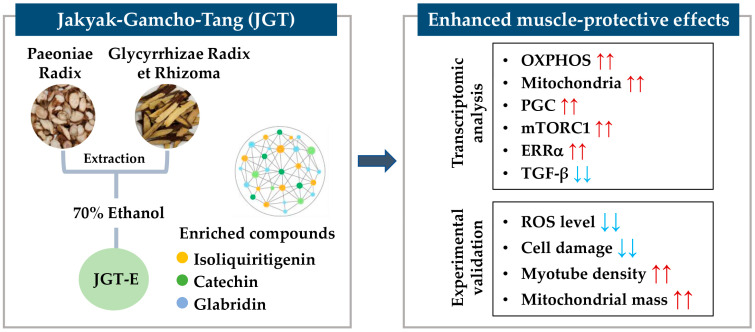
Schematic summary of the muscle-protective mechanism of JGT-E. Jakyak-Gamcho-Tang (JGT), a traditional herbal formulation consisting of Paeoniae Radix and Glycyrrhizae Radix et Rhizoma, was extracted using 70% ethanol to yield JGT-E, which is enriched in key bioactive compounds including isoliquiritigenin, catechin, and glabridin. Transcriptomic analysis revealed that JGT-E significantly upregulated genes associated with oxidative phosphorylation (OXPHOS), mitochondrial biogenesis, PGC-1α, mTORC1, and ERRα signaling, while downregulating TGF-β signaling. These molecular changes were validated experimentally and were accompanied by decreased intracellular ROS levels and cell damage, as well as increased mitochondrial content and myotube integrity. Collectively, these findings support the enhanced muscle-protective efficacy of JGT-E. Red arrows indicate upregulation, and blue arrows indicate downregulation.

This study demonstrated that JGT-E provides superior protection against muscle atrophy compared to JGT-W, largely due to its higher contents of key bioactive compounds such as isoliquiritigenin, catechin, and glabridin. Through an integrative approach combining transcriptome analysis with network pharmacology, we showed that JGT-E enhances mitochondrial biogenesis, oxidative phosphorylation, and anabolic signaling more effectively, while simultaneously suppressing muscle-atrophic TGF-β pathways. Functional assays using C2C12 mouse muscle cells confirmed that JGT-E significantly reduces oxidative stress and preserves myotube integrity under stress conditions, outperforming JGT-W (Figure 7). These findings highlight the importance of integrating systems-level analysis with experimental validation and emphasize the critical role of extraction methods in optimizing the therapeutic efficacy of herbal medicines for treating muscle atrophy.

## Figures and Tables

**Table 1:** The condition of MRM for UHPLC-TQ-MS/MS.

Peak No.	Components	Rt (min)	Precursor Ion (*m*/*z*)	Product Ion (*m*/*z*)	Collision Energy (V)	Ion Polarity
1	Oxypaeoniflorin	2.985	495.1	137.1	26.0	Negative
2	(+)-Catechin	3.115	291.1	139.0	14.0	Positive
3	Albiflorin	3.566	481.2	105.0	22.0	Positive
4	Paeoniflorin	3.759	525.2	449.1	10.0	Negative
5	Liquiritin apioside	4.341	549.2	255.1	34.0	Negative
6	Liquiritin	4.535	417.2	255.0	20.0	Negative
7	1,2,3,4,6-Pentagalloyl glucose	4.680	958.1	153.0	40.0	Positive
8	Isoliquiritin apioside	7.169	549.1	255.1	30.0	Negative
9	Ononin	7.634	431.1	269.1	18.0	Positive
10	Isoliquiritin	7.789	417.0	255.1	18.0	Negative
11	Liquiritigenin	9.025	257.0	137.0	26.0	Positive
12	Benzoylpaeoniflorin	11.531	629.1	553.2	10.0	Negative
13	Isoliquiritigenin	13.811	257.0	137.0	22.0	Positive
14	Glycyrrhizin	15.030	821.4	351.0	40.0	Negative
15	Glabridin	19.529	325.1	189.1	14.0	Positive
16	Glycyrrhetinic acid	21.004	471.2	189.1	38.0	Positive

**Table 2:** The linearity, range, and contents of 16 components in JGT-W and JGT-E using UHPLC-MS/MS analysis.

No.	Components	Regression Equation	R^2^	Range (μg/mL)	Concentration (mg/g, *n* = 6)	
					JGT-W	JGT-E
1	Oxypaeoniflorin	y = 86,522x + 6367	0.9990	0.16–2.50	0.959 (±0.024)	0.875 (±0.012)
2	(+)-Catechin	y = 484,425x + 23,507	0.9990	0.08–2.5	0.089 (±0.003)	1.101 (±0.016)
3	Albiflorin	y = 590,324x + 8051	0.9992	0.04–5.00	15.141 (±1.636)	14.881 (±0.675)
4	Paeoniflorin	y = 205,795x + 93,092	0.9990	0.63–10.00	66.180 (±6.085)	70.331 (±3.824)
5	Liquiritin apioside	y = 379,464x + 22,860	0.9991	0.04–2.50	9.013 (±0.931)	10.271 (±0.748)
6	Liquiritin	y = 459,755x + 37,366	0.9990	0.08–1.25	5.158 (±0.461)	6.956 (±0.584)
7	1,2,3,4,6-Pentagalloyl glucose	y = 220,570x + 3267	0.9991	0.04–5.00	0.295 (±0.022)	2.240 (±0.047)
8	Isoliquiritin apioside	y = 162,498x + 80,392	0.9991	0.63–10.00	0.165 (±0.013)	6.092 (±0.066)
9	Ononin	y = 2,566,720x + 52,6143	0.9991	0.31–5.00	3.896 (±0.039)	4.234 (±0.069)
10	Isoliquiritin	y = 406,963x + 15,5124	0.9991	0.31–5.00	tr *	1.849 (±0.029)
11	Liquiritigenin	y = 1,525,781x + 42,803	0.9992	0.04–1.25	tr	1.038 (±0.013)
12	Benzoylpaeoniflorin	y = 306,585x + 50,791	0.9990	0.16–5.00	0.643 (±0.015)	0.975 (±0.013)
13	Isoliquiritigenin	y = 1,244,869x + 29,884	0.9992	0.04–2.50	0.006 (±0.002)	1.184 (±0.017)
14	Glycyrrhizin	y = 80,641x + 18,808	0.9990	0.16–10.00	50.324 (±4.754)	75.053 (±4.004)
15	Glabridin	y = 1,288,421x + 10,894	0.9996	0.04–0.63	0.022 (±0.001)	0.838 (±0.077)
16	Glycyrrhetinic acid	y = 558,961x + 28,138	0.9990	0.08–1.25	tr	0.241 (±0.004)

* tr; trace.

## Data Availability

The raw sequencing data (FASTQ files) and processed count data were uploaded to the Gene Expression Omnibus under the accession number GSE295069 (reviewer’s access link: https://www.ncbi.nlm.nih.gov/geo/query/acc.cgi?acc=GSE295069 (accessed on 25 June 2025).

## Supplementary Materials

The following supporting information can be downloaded at: https://www.mdpi.com/article/10.3390/antiox14070795/s1. Figure S1: Transcriptomic effects regulated by JGT-E and JGT-W, Figure S2: Effects of JGT-W, JGT-E, resveratrol, and arachidonic acid under conditions of muscle atrophy in C2C12 myotubes, Figure S3: Dynamic multiple reaction monitoring (MRM) mode of JGT-W (A), JGT-E (B), and standard mixture (C) using a UHPLC-TQ-MS/MS, Figure S4: Muscle-protective activity of 16 compounds in JGT, Table S1: Recovery data of the 16 compounds in JGT.

## Abbreviations

The following abbreviations are used in this manuscript:

JGT	Jakyak-gamcho-tang
ABTS	2,2′-Azino-bis(3-ethylbenzothiazoline-6-sulfonic acid) diammonium salt
BPC	Base peak ion chromatogram
DEG	Differentially expressed genes
DM	Differentiation medium
DMEM	Dulbecco’s Modified Eagle Medium
DMSO	Dimethyl sulfoxide
DPPH	2,2-diphenyl-1-picrylhydrazyl
FDR	False discovery rate
GM	Growth medium
GO	Gene Ontology
GOBP	Gene Ontology Biological Process
GSEA	Gene Set Enrichment Analysis
H_2_O_2_	Hydrogen peroxide
JGT	Jakyak-gamcho-tang
JGT-E	Ethanol extract of Jakyak-gamcho-tang
JGT-W	Water extract of Jakyak-gamcho-tang
MRM	Multiple reaction monitoring
NES	Normalized enrichment score
OD	Optical density
PBS	Phosphate-buffered saline
P/S	Penicillin and streptomycin
ROS	Reactive oxygen species
SD	Standard deviation
SDS	Sodium dodecyl sulfate
UHPLC	Ultrahigh-performance liquid chromatography

## References

[1] Cohen S., Nathan J.A., Goldberg A.L. (2015). Muscle wasting in disease: Molecular mechanisms and promising therapies. Nat. Rev. Drug. Discov..

[2] Yin L., Li N., Jia W., Wang N., Liang M., Yang X., Du G. (2021). Skeletal muscle atrophy: From mechanisms to treatments. Pharmacol. Res..

[3] Gadelha A.B., Neri S.G.R., Oliveira R.J., Bottaro M., David A.C., Vainshelboim B., Lima R.M. (2018). Severity of sarcopenia is associated with postural balance and risk of falls in community-dwelling older women. Exp. Aging Res..

[4] Yadav A., Yadav S.S., Singh S., Dabur R. (2022). Natural products: Potential therapeutic agents to prevent skeletal muscle atrophy. Eur. J. Pharmacol..

[5] Jung H.W., Kang A.N., Kang S.Y., Park Y.K., Song M.Y. (2017). The root extract of *Pueraria lobata* and its main compound, puerarin, prevent obesity by increasing the energy metabolism in skeletal muscle. Nutrients.

[6] Ota K., Fukui K., Nakamura E., Oka M., Ota K., Sakaue M., Sano Y., Takasu A. (2020). Effect of Shakuyaku-kanzo-to in patients with muscle cramps: A systematic literature review. J. Gen. Fam. Med..

[7] Kim A., Kim Y.R., Park S.M., Lee H., Park M., Yi J.M., Cha S., Kim N.S. (2024). Jakyak-gamcho-tang, a decoction of Paeoniae Radix and Glycyrrhizae Radix et Rhizoma, ameliorates dexamethasone-induced muscle atrophy and muscle dysfunction. Phytomedicine.

[8] Abubakar A.R., Haque M. (2020). Preparation of medicinal plants: Basic extraction and fractionation procedures for experimental purposes. J. Pharm. Bioallied Sci..

[9] Plaskova A., Mlcek J. (2023). New insights of the application of water or ethanol-water plant extract rich in active compounds in food. Front. Nutr..

[10] Stanciauskaite M., Marksa M., Babickaite L., Majiene D., Ramanauskiene K. (2021). Comparison of ethanolic and aqueous *Populus balsamifera* L. Bud Extracts by different extraction methods: Chemical composition, antioxidant and antibacterial activities. Pharmaceuticals.

[11] Ju J.B., Kim J.S., Choi C.W., Lee H.K., Oh T.K., Kim S.C. (2008). Comparison between ethanolic and aqueous extracts from Chinese juniper berries for hypoglycaemic and hypolipidemic effects in alloxan-induced diabetic rats. J. Ethnopharmacol..

[12] Borges A., Jose H., Homem V., Simoes M. (2020). Comparison of techniques and solvents on the antimicrobial and antioxidant potential of extracts from *Acacia dealbata* and *Olea europaea*. Antibiotics.

[13] Bae J.Y., Lee Y.S., Han S.Y., Jeong E.J., Lee M.K., Kong J.Y., Lee D.H., Cho K.J., Lee H.S., Ahn M.J. (2012). A Comparison between water and ethanol extracts of *Rumex acetosa* for protective effects on gastric ulcers in mice. Biomol. Ther..

[14] Park S.M., Kim A., Lee H., Baek S.J., Kim N.S., Park M., Yi J.M., Cha S. (2022). Systematic transcriptome analysis reveals molecular mechanisms and indications of bupleuri radix. Front. Pharmacol..

[15] Park M., Park S.M., Lee H., Kim A., Kim N.S., Kim Y.R., Yi J.M., Cha S. (2024). KORE-Map 1.0: Korean Medicine Omics Resource Extension Map on transcriptome data of tonifying herbal medicine. Sci. Data.

[16] Kim Y.S., Yuk H.J., Kim D.S. (2021). Effect of Jakyakgamcho-Tang extracts on H_2_O_2_-induced C2C12 myoblasts. Molecules.

[17] Zhang G.B., Li Q.Y., Chen Q.L., Su S.B. (2013). Network pharmacology: A new approach for chinese herbal medicine research. Evid. Based Complement. Alternat. Med..

[18] Noor F., Tahir Ul Qamar M., Ashfaq U.A., Albutti A., Alwashmi A.S.S., Aljasir M.A. (2022). Network pharmacology approach for medicinal plants: Review and assessment. Pharmaceuticals.

[19] Choi W.G., Choi N.R., Park E.J., Kim B.J. (2022). A study of the therapeutic mechanism of Jakyakgamcho-Tang about functional dyspepsia through network pharmacology research. Int. J. Med. Sci..

[20] Hu Y.H., Wang X.Y., Zhang X.W., Chen J., Li F. (2022). Investigation of the mechanisms and experimental verification of Shao yao gan cao decoction against Sphincter of Oddi Dysfunction *via* systems pharmacology. Math. Biosci. Eng..

[21] Alamdari N., Aversa Z., Castillero E., Gurav A., Petkova V., Tizio S., Hasselgren P.O. (2012). Resveratrol prevents dexamethasone-induced expression of the muscle atrophy-related ubiquitin ligases atrogin-1 and MuRF1 in cultured myotubes through a SIRT1-dependent mechanism. Biochem. Biophys. Res. Commun..

[22] Bagherniya M., Mahdavi A., Shokri-Mashhadi N., Banach M., Von Haehling S., Johnston T.P., Sahebkar A. (2022). The beneficial therapeutic effects of plant-derived natural products for the treatment of sarcopenia. J. Cachexia Sarcopenia Muscle.

[23] Mitra A., Shanavas S., Chaudhury D., Bose B., Das U.N., Shenoy P.S. (2023). Mitigation of chronic glucotoxicity-mediated skeletal muscle atrophy by arachidonic acid. Life Sci..

[24] Chen X., Ji Y., Liu R., Zhu X., Wang K., Yang X., Liu B., Gao Z., Huang Y., Shen Y. (2023). Mitochondrial dysfunction: Roles in skeletal muscle atrophy. J. Transl. Med..

[25] Popov L.D. (2020). Mitochondrial biogenesis: An update. J. Cell. Mol. Med..

[26] Sandri M., Lin J., Handschin C., Yang W., Arany Z.P., Lecker S.H., Goldberg A.L., Spiegelman B.M. (2006). PGC-1alpha protects skeletal muscle from atrophy by suppressing FoxO3 action and atrophy-specific gene transcription. Proc. Natl. Acad. Sci. USA.

[27] Handschin C., Spiegelman B.M. (2008). The role of exercise and PGC-1α in inflammation and chronic disease. Nature.

[28] Gatica D., Klionsky D.J. (2017). New insights into MTORC1 amino acid sensing and activation. Biotarget.

[29] Rudrappa S.S., Wilkinson D.J., Greenhaff P.L., Smith K., Idris I., Atherton P.J. (2016). Human skeletal muscle disuse atrophy: Effects on muscle protein synthesis, breakdown, and insulin resistance-A qualitative review. Front. Physiol..

[30] Mendias C.L., Gumucio J.P., Davis M.E., Bromley C.W., Davis C.S., Brooks S.V. (2012). Transforming growth factor-beta induces skeletal muscle atrophy and fibrosis through the induction of atrogin-1 and scleraxis. Muscle Nerve.

[31] Gurd B.J. (2011). Deacetylation of PGC-1alpha by SIRT1: Importance for skeletal muscle function and exercise-induced mitochondrial biogenesis. Appl. Physiol. Nutr. Metab..

[32] Schreiber S.N., Emter R., Hock M.B., Knutti D., Cardenas J., Podvinec M., Oakeley E.J., Kralli A. (2004). The estrogen-related receptor alpha (ERRalpha) functions in PPARgamma coactivator 1alpha (PGC-1alpha)-induced mitochondrial biogenesis. Proc. Natl. Acad. Sci. USA.

[33] Sandri M., Sandri C., Gilbert A., Skurk C., Calabria E., Picard A., Walsh K., Schiaffino S., Lecker S.H., Goldberg A.L. (2004). Foxo transcription factors induce the atrophy-related ubiquitin ligase atrogin-1 and cause skeletal muscle atrophy. Cell.

[34] Shi D., Yang J., Jiang Y., Wen L., Wang Z., Yang B. (2020). The antioxidant activity and neuroprotective mechanism of isoliquiritigenin. Free Radic. Biol. Med..

[35] Wang T.T., Chen Z.Z., Xie P., Zhang W.J., Du M.Y., Liu Y.T., Zhu H.Y., Guo Y.S. (2019). Isoliquiritigenin suppresses the proliferation and induced apoptosis *via* miR-32/LATS2/Wnt in nasopharyngeal carcinoma. Eur. J. Pharmacol..

[36] Al-Qahtani W.H., Alshammari G.M., Ajarem J.S., Al-Zahrani A.Y., Alzuwaydi A., Eid R., Yahya M.A. (2022). Isoliquiritigenin prevents doxorubicin-induced hepatic damage in rats by upregulating and activating SIRT1. Biomed. Pharmacother..

[37] Higdon J.V., Frei B. (2003). Tea catechins and polyphenols: Health effects, metabolism, and antioxidant functions. Crit. Rev. Food Sci. Nutr..

[38] Zhang J., Wu X., Zhong B., Liao Q., Wang X., Xie Y., He X. (2023). Review on the diverse biological effects of glabridin. Drug Des. Devel. Ther..

[39] Denzer I., Munch G., Pischetsrieder M., Friedland K. (2016). S-allyl-L-cysteine and isoliquiritigenin improve mitochondrial function in cellular models of oxidative and nitrosative stress. Food Chem..

[40] Yang E.J., Min J.S., Ku H.Y., Choi H.S., Park M.K., Kim M.K., Song K.S., Lee D.S. (2012). Isoliquiritigenin isolated from *Glycyrrhiza uralensis* protects neuronal cells against glutamate-induced mitochondrial dysfunction. Biochem. Biophys. Res. Commun..

[41] Jiao X., Zhang Y., Wang Z., Chang H., Li Y., Wang B., Gan Y., Gu D. (2025). Isoliquiritigenin, an extract from Licorice, attenuates dexamethasone-induced muscle atrophy *via* Akt/mTOR pathway. Mol. Nutr. Food Res..

[42] Li P., Liu A., Xiong W., Lin H., Xiao W., Huang J., Zhang S., Liu Z. (2020). Catechins enhance skeletal muscle performance. Crit. Rev. Food Sci. Nutr..

[43] Kim A.R., Kim K.M., Byun M.R., Hwang J.H., Park J.I., Oh H.T., Kim H.K., Jeong M.G., Hwang E.S., Hong J.H. (2017). Catechins activate muscle stem cells by Myf5 induction and stimulate muscle regeneration. Biochem. Biophys. Res. Commun..

[44] Li P., Liu A., Liu C., Qu Z., Xiao W., Huang J., Liu Z., Zhang S. (2019). Role and mechanism of catechin in skeletal muscle cell differentiation. J. Nutr. Biochem..

[45] Yoshioka Y., Kubota Y., Samukawa Y., Yamashita Y., Ashida H. (2019). Glabridin inhibits dexamethasone-induced muscle atrophy. Arch. Biochem. Biophys..

