# Supplementary material for: Integration of Transcriptomic Analysis, Network Pharmacology, and Experimental Validation Demonstrates Enhanced Muscle-Protective Effects of Ethanol Extract of Jakyak-Gamcho-Tang
Source: Antioxidants (Basel). 2025 Jun 27;14(7):795. doi: 10.3390/antiox14070795 (PMC12291634; doi:10.3390/antiox14070795)
Supplement: Supplementary file 1 [file antioxidants-14-00795-s001.zip › antioxidants-3660629-supplementary.pdf]

Supplementary Materials for

Integration of transcriptomic analysis, network pharmacology, and experimental validation demonstrates enhanced muscle-protective effects of ethanol extract of Jakyak-gamcho-tang

Aeyung Kim[1,*,+], Minh Nhat Tran[2,3,+], A Yeong Lee[4], Heerim Yeo[2], Su-Jin Baek[4], No Soo Kim[5], Seongwon Cha[4], Sang-Min Park[2,*]

[1]KM Application Center, Korea Institute of Oriental Medicine, Daegu, Republic of Korea; aykim71@kiom.re.kr (A.K.)

[2]College of Pharmacy, Chungnam National University, Daejeon, Republic of Korea; tnminh@huemed-univ.edu.vn (M.N.T.), yeooh1@naver.com (H.Y.), smpark@cnu.ac.kr (S.-M.P.)

[3]Faculty of Traditional Medicine, Hue University of Medicine and Pharmacy, Hue University, Hue, Viet Nam; tnminh@huemed-univ.edu.vn (M.N.T.)

[4]KM Data Division, Korea Institute of Oriental Medicine, Daejeon, Republic of Korea; lay7709@kiom.re.kr (A.Y.L.), baeksj@kiom.re.kr (S.-J.B.), scha@kiom.re.kr (S.C.)

[5]KM Convergence Research Division, Korea Institute of Oriental Medicine, Daejeon, Republic of Korea; nosookim@kiom.re.kr (N.S.K.)

[+]These authors contributed equally to this work.

[*]Correspondence: aykim71@kiom.re.kr (A.K.); smpark@cnu.ac.kr (S-M.P.); Tel.: +82-53-940-3830 (A.K.); +82-42-821-5919 (S-M.P.)

This PDF file includes: Figure S1-S4 and Table S1

Figure S1

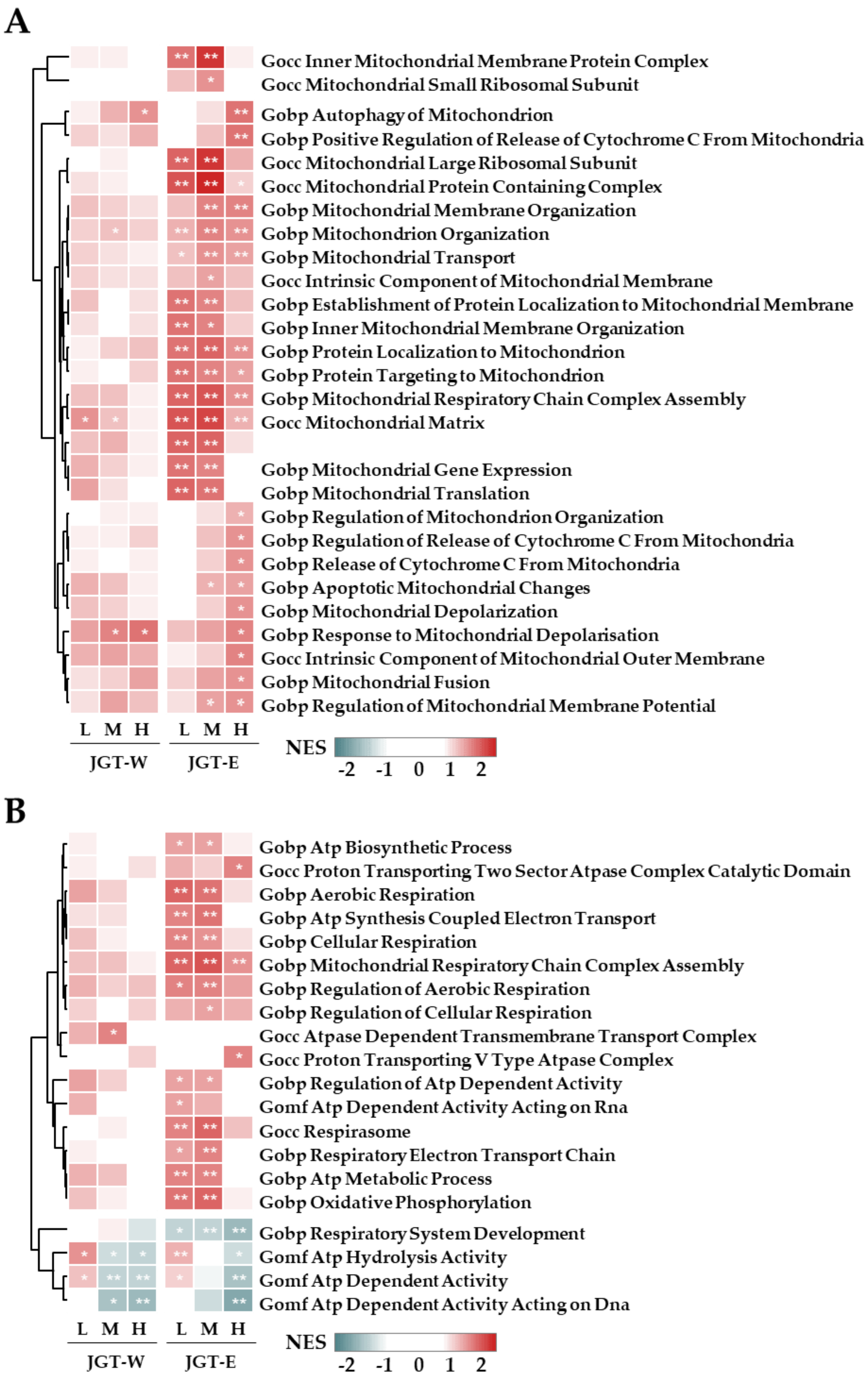
A
Gocc Inner Mitochondrial Membrane Protein Complex
Gocc Mitochondrial Small Ribosomal Subunit
Gobp Autophagy of Mitochondrion
Gobp Positive Regulation of Release of Cytochrome C From Mitochondria
Gocc Mitochondrial Large Ribosomal Subunit
Gocc Mitochondrial Protein Containing Complex
Gobp Mitochondrial Membrane Organization
Gobp Mitochondrion Organization
Gobp Mitochondrial Transport
Gocc Intrinsic Component of Mitochondrial Membrane
Gobp Establishment of Protein Localization to Mitochondrial Membrane
Gobp Inner Mitochondrial Membrane Organization
Gobp Protein Localization to Mitochondrion
Gobp Protein Targeting to Mitochondrion
Gobp Mitochondrial Respiratory Chain Complex Assembly
Gocc Mitochondrial Matrix
Gobp Mitochondrial Gene Expression
Gobp Mitochondrial Translation
Gobp Regulation of Mitochondrion Organization
Gobp Regulation of Release of Cytochrome C From Mitochondria
Gobp Release of Cytochrome C From Mitochondria
Gobp Apoptotic Mitochondrial Changes
Gobp Mitochondrial Depolarization
Gobp Response to Mitochondrial Depolarisation
Gocc Intrinsic Component of Mitochondrial Outer Membrane
Gobp Mitochondrial Fusion
Gobp Regulation of Mitochondrial Membrane Potential
L M H
JGT-W
L M H
JGT-E
NES
-2 -1 0 1 2
B
Gobp Atp Biosynthetic Process
Gocc Proton Transporting Two Sector Atpase Complex Catalytic Domain
Gobp Aerobic Respiration
Gobp Atp Synthesis Coupled Electron Transport
Gobp Cellular Respiration
Gobp Mitochondrial Respiratory Chain Complex Assembly
Gobp Regulation of Aerobic Respiration
Gobp Regulation of Cellular Respiration
Gocc Atpase Dependent Transmembrane Transport Complex
Gocc Proton Transporting V Type Atpase Complex
Gobp Regulation of Atp Dependent Activity
Gomf Atp Dependent Activity Acting on Rna
Gocc Respirasome
Gobp Respiratory Electron Transport Chain
Gobp Atp Metabolic Process
Gobp Oxidative Phosphorylation
Gobp Respiratory System Development
Gomf Atp Hydrolysis Activity
Gomf Atp Dependent Activity
Gomf Atp Dependent Activity Acting on Dna
L M H
JGT-W
L M H
JGT-E
NES
-2 -1 0 1 2

Figure S1

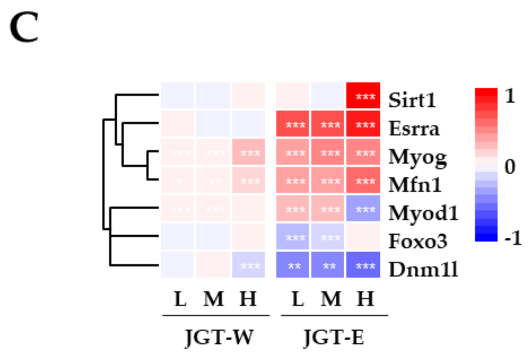

Figure S1. Transcriptomic effects regulated by JGT-E and JGT-W. **(A–B)** Gene Set Enrichment Analysis (GSEA) results of C2C12 myotubes treated with JGT-E and JGT-W at low (L, 20 μg/mL), medium (M, 100 μg/mL), and high (H, 500 μg/mL) doses. Gene ontology (GO) gene sets were categorized into biological process (GOBP), molecular function (GOMF), and cellular component (GOCC). Panels **(A)** and **(B)** display keyword-filtered pathways, with panel **(A)** highlighting mitochondrial-related pathways and panel **(B)** focusing on those involved in cellular respiration and ATP production. Normalized enrichment scores (NES) are represented by color intensity. **(C)** The $\log_2$ fold change ($\log_2$FC) values of representative genes associated with mitochondrial biogenesis and myogenic differentiation.

Figure S2

A

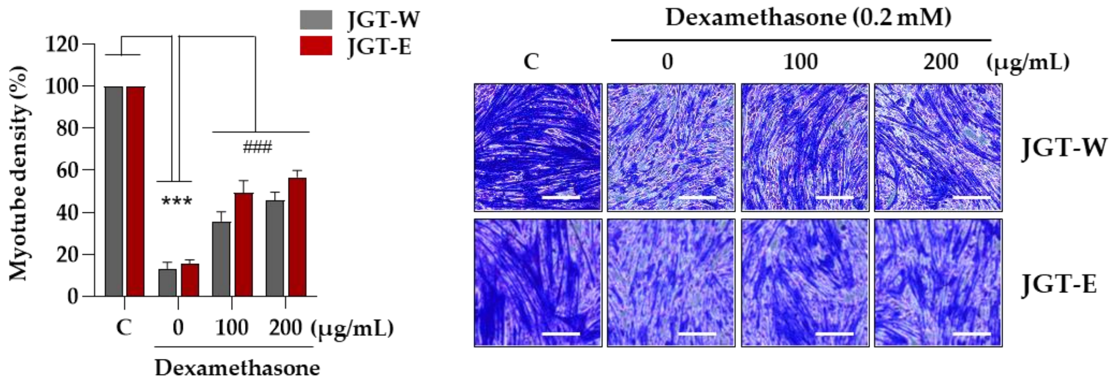

JGT-W
JGT-E
Myotube density (%)
120
100
80
60
40
20
0
***
###
C
0
100
200 (μg/mL)
Dexamethasone
Dexamethasone (0.2 mM)
C
0
100
200 (μg/mL)
JGT-W
JGT-E

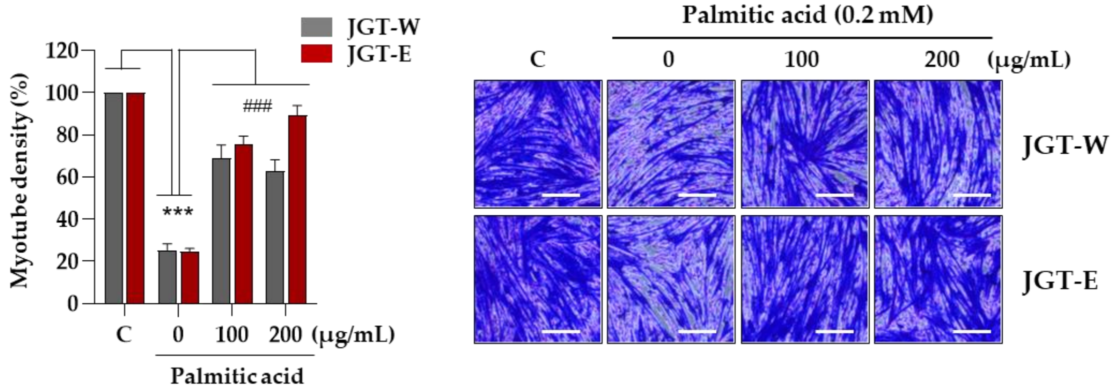

JGT-W
JGT-E
Myotube density (%)
120
100
80
60
40
20
0
***
###
C
0
100
200 (μg/mL)
Palmitic acid
Palmitic acid (0.2 mM)
C
0
100
200 (μg/mL)
JGT-W
JGT-E

B

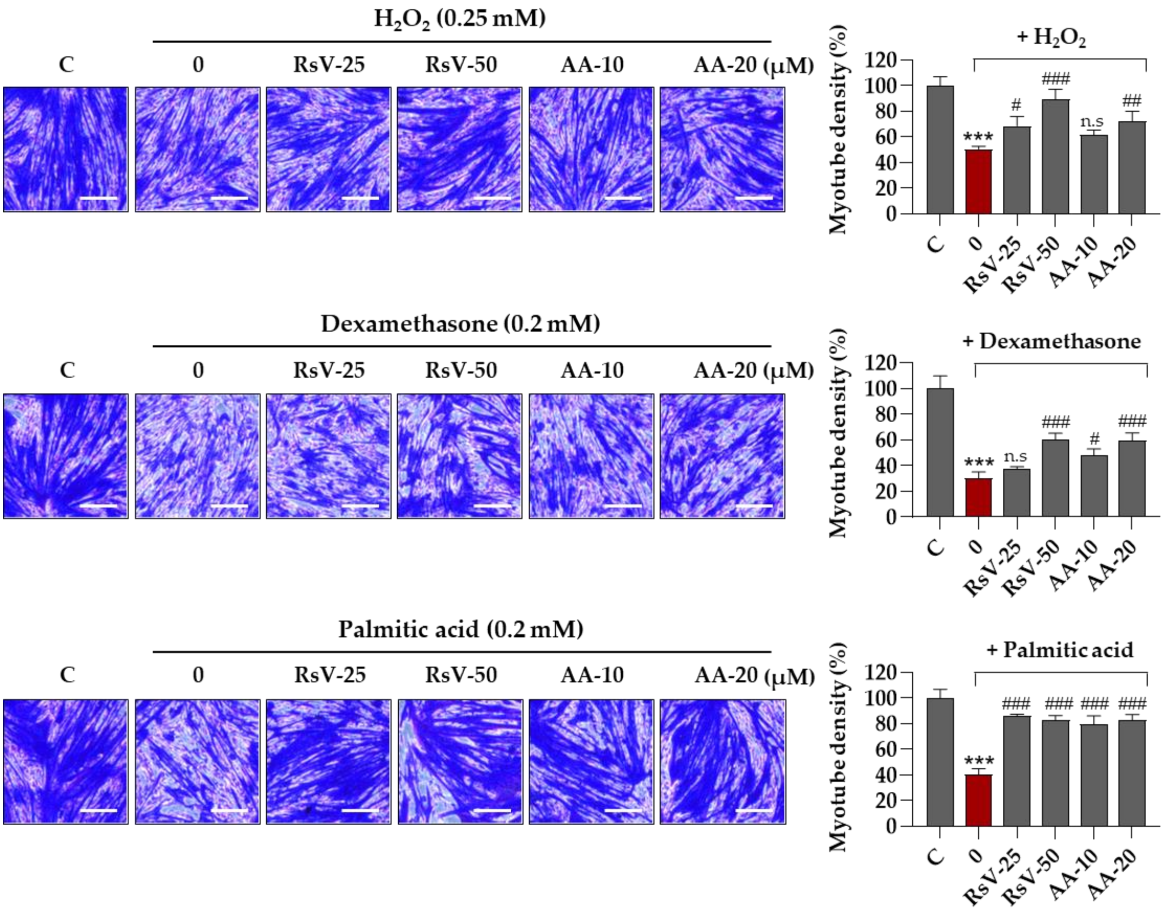

Figure S2. Effects of JGT-W, JGT-E, resveratrol and arachidonic acid under conditions of muscle atrophy in C2C12 myotubes. **(A)** C2C12 myotubes were pretreated with 100 and 200 μg/mL of JGT-W and JGT-E for 12 h and subsequently exposed to 0.2 mM dexamethasone or 0.2 mM palmitic acid for an additional 40 h. **(B)** C2C12 myotubes were pretreated with indicated concentrations of resveratrol (RsV) and arachidonic acid (AA) for 12 h and subsequently exposed to 0.25 mM H_2O_2, 0.2 mM dexamethasone or 0.2 mM palmitic acid for an additional 40 h. Myotube density was quantified through crystal violet staining. To Data are presented as the mean ± SD (n = 3). Statistical significance was evaluated by one-way ANOVA followed by Dunnett's multiple comparison test. ***p < 0.001 vs. vehicle-treated controls; #p < 0.05, #p < 0.01, ###p < 0.001 vs. H_2O_2, dexamethasone or palmitic acid + vehicle-treated control cells. Scale bar = 100 μm.

Figure S3

A

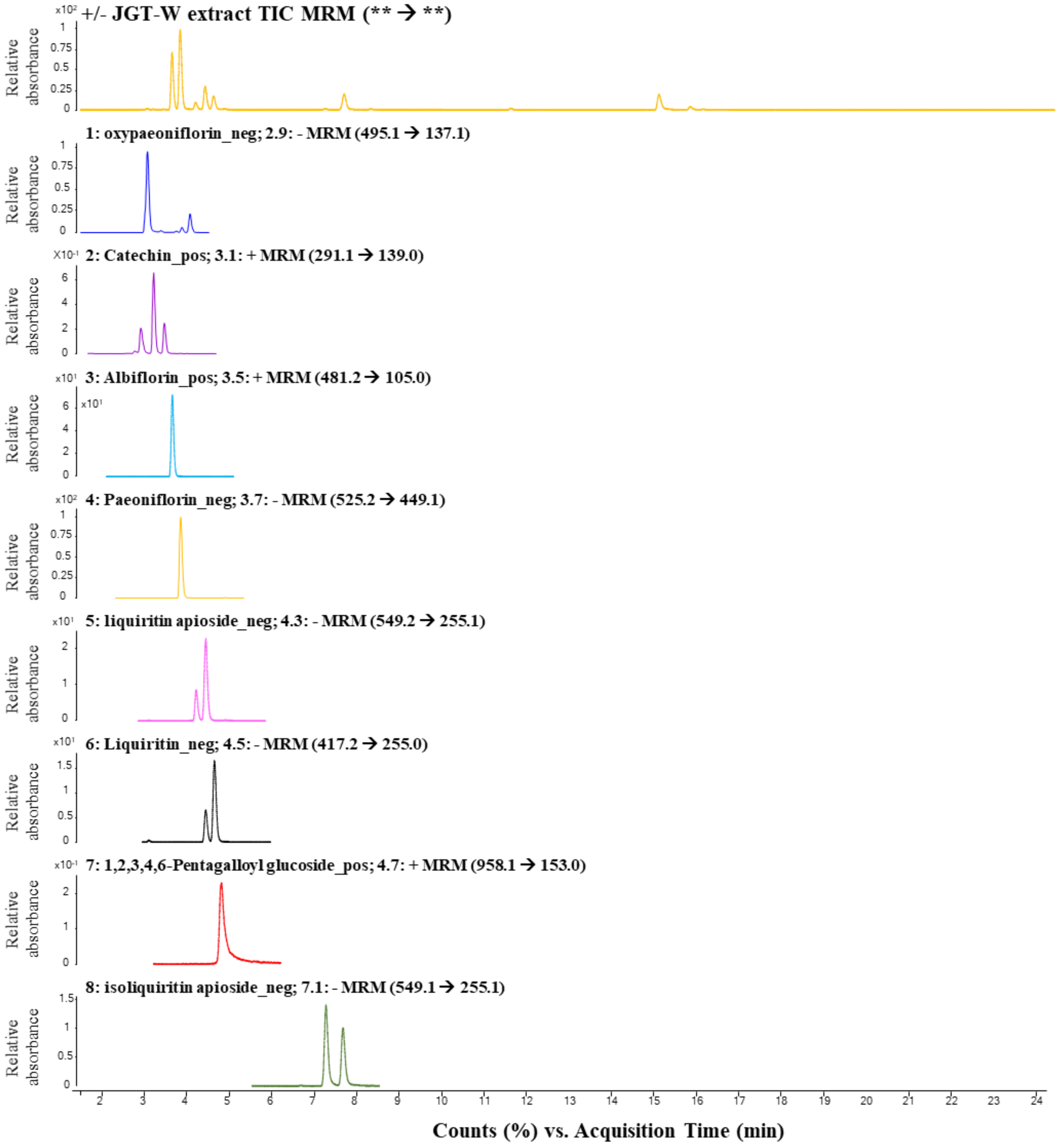

+/- JGT-W extract TIC MRM (** → **)
1: oxypaeoniflorin_neg; 2.9: - MRM (495.1 → 137.1)
2: Catechin_pos; 3.1: + MRM (291.1 → 139.0)
3: Albiflorin_pos; 3.5: + MRM (481.2 → 105.0)
4: Paeoniflorin_neg; 3.7: - MRM (525.2 → 449.1)
5: liquiritin apioside_neg; 4.3: - MRM (549.2 → 255.1)
6: Liquiritin_neg; 4.5: - MRM (417.2 → 255.0)
7: 1,2,3,4,6-Pentagalloyl glucoside_pos; 4.7: + MRM (958.1 → 153.0)
8: isoliquiritin apioside_neg; 7.1: - MRM (549.1 → 255.1)
Relative absorbance
Counts (%) vs. Acquisition Time (min)

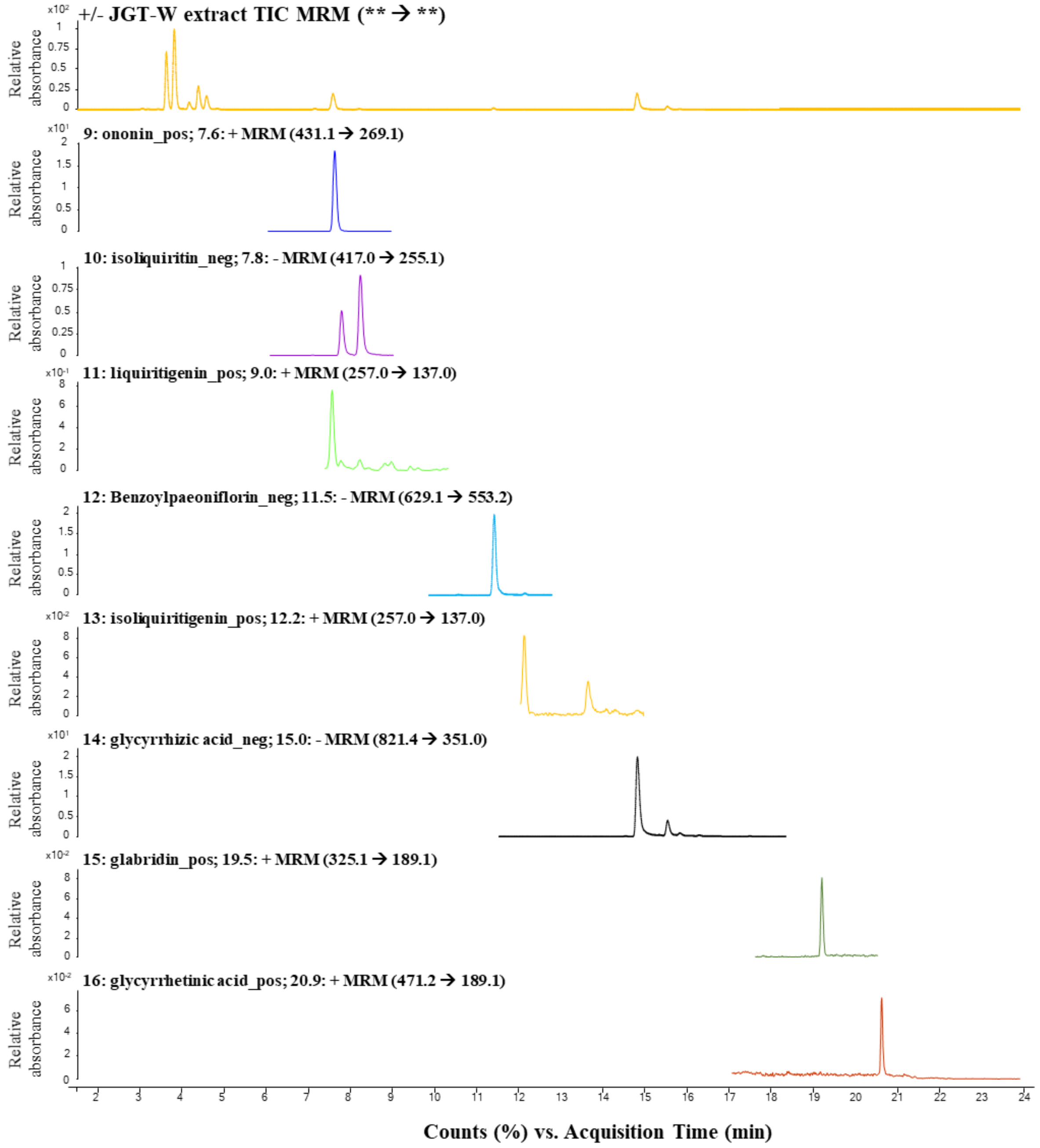
+/- JGT-W extract TIC MRM (** → **)
9: ononin_pos; 7.6: + MRM (431.1 → 269.1)
10: isoliquiritin_neg; 7.8: - MRM (417.0 → 255.1)
11: liquiritigenin_pos; 9.0: + MRM (257.0 → 137.0)
12: Benzoylpaeoniflorin_neg; 11.5: - MRM (629.1 → 553.2)
13: isoliquiritigenin_pos; 12.2: + MRM (257.0 → 137.0)
14: glycyrrhizic acid_neg; 15.0: - MRM (821.4 → 351.0)
15: glabridin_pos; 19.5: + MRM (325.1 → 189.1)
16: glycyrrhetinic acid_pos; 20.9: + MRM (471.2 → 189.1)
Relative absorbance
Counts (%) vs. Acquisition Time (min)

B

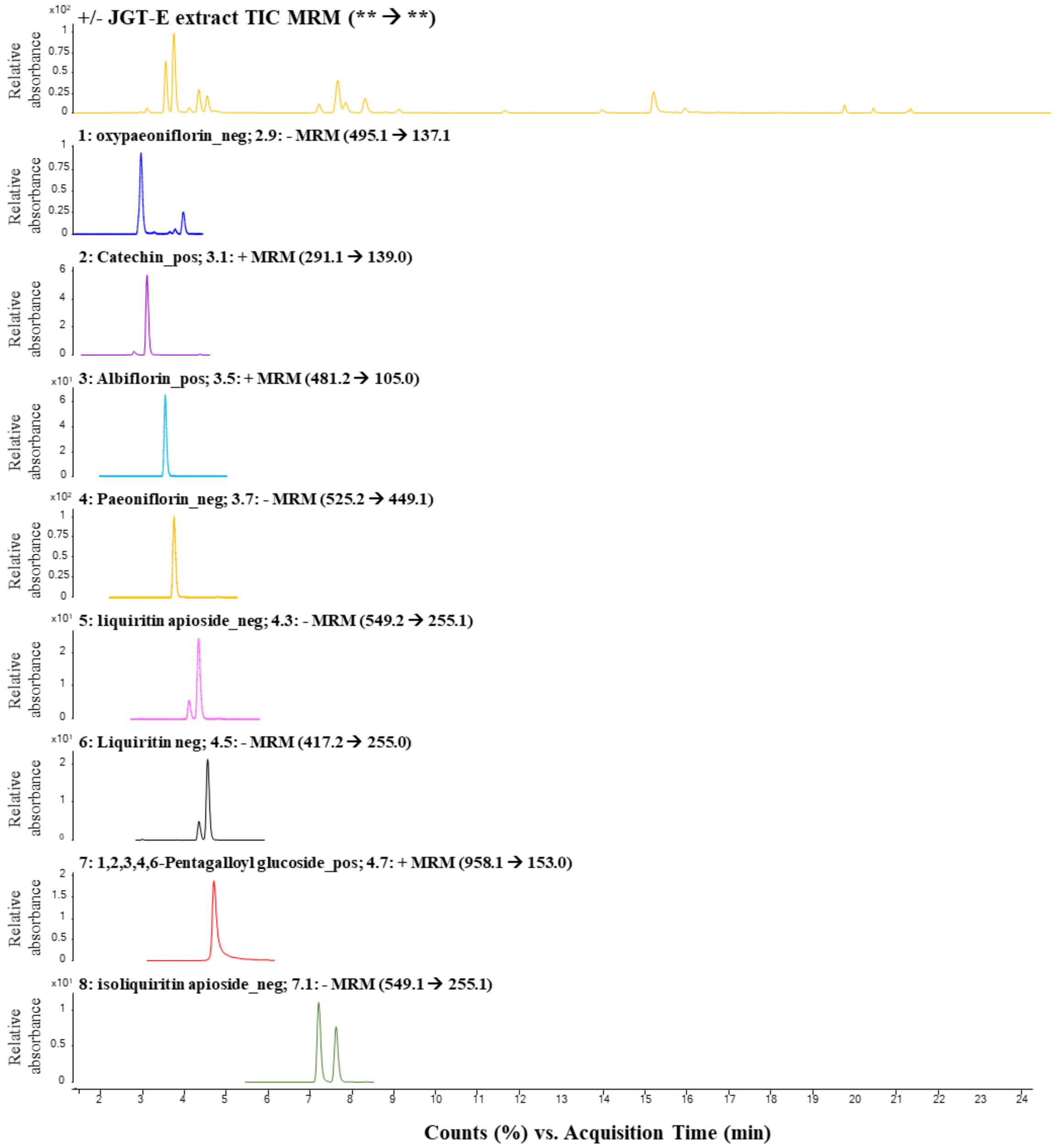
+/- JGT-E extract TIC MRM (** → **)
1: oxypaeoniflorin_neg; 2.9: - MRM (495.1 → 137.1
2: Catechin_pos; 3.1: + MRM (291.1 → 139.0)
3: Albiflorin_pos; 3.5: + MRM (481.2 → 105.0)
4: Paeoniflorin_neg; 3.7: - MRM (525.2 → 449.1)
5: liquiritin apioside_neg; 4.3: - MRM (549.2 → 255.1)
6: Liquiritin neg; 4.5: - MRM (417.2 → 255.0)
7: 1,2,3,4,6-Pentagalloyl glucoside_pos; 4.7: + MRM (958.1 → 153.0)
8: isoliquiritin apioside_neg; 7.1: - MRM (549.1 → 255.1)
Relative absorbance
Counts (%) vs. Acquisition Time (min)

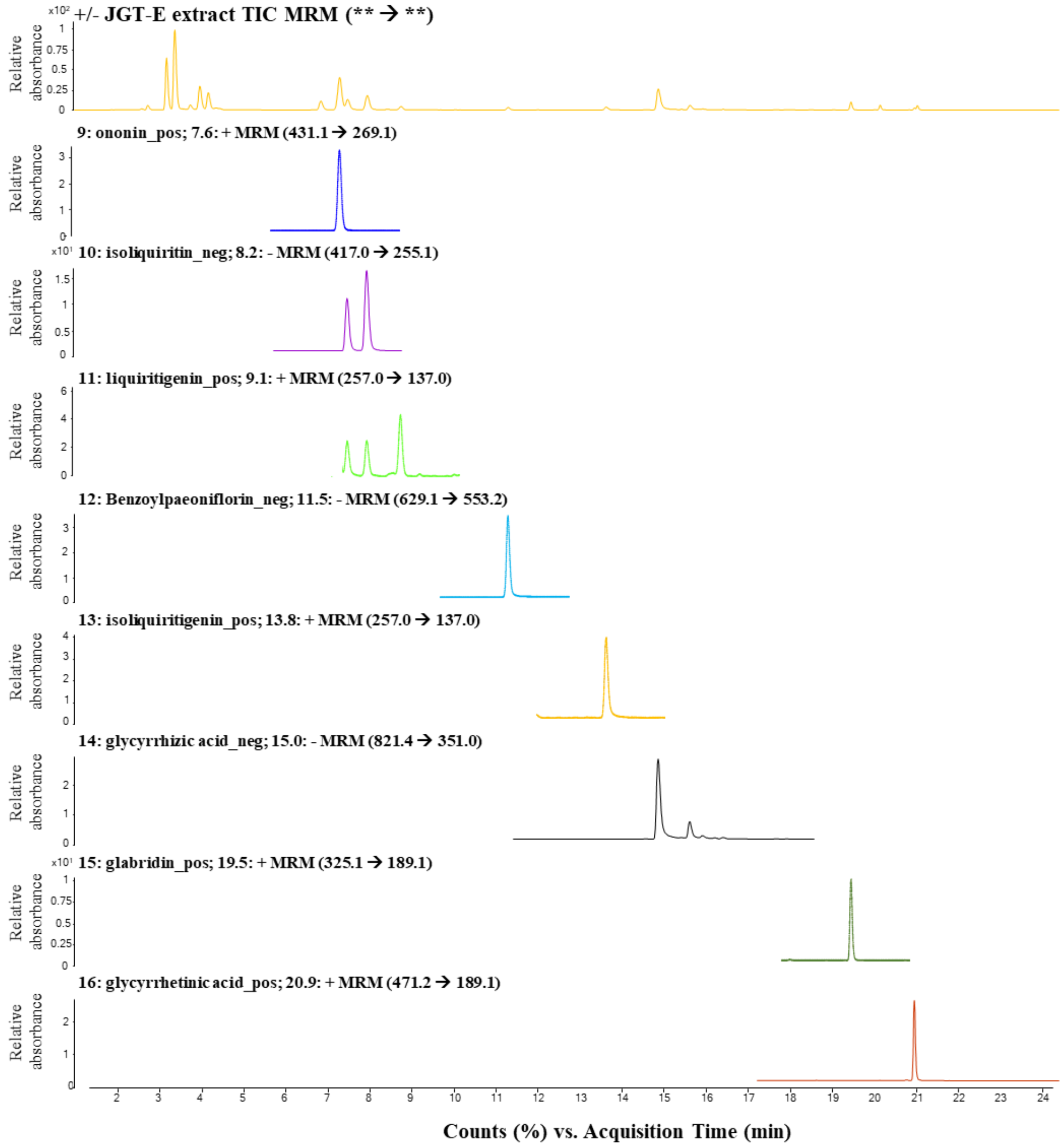

+/- JGT-E extract TIC MRM (** → **)
9: ononin_pos; 7.6: + MRM (431.1 → 269.1)
10: isoliquiritin_neg; 8.2: - MRM (417.0 → 255.1)
11: liquiritigenin_pos; 9.1: + MRM (257.0 → 137.0)
12: Benzoylpaeoniflorin_neg; 11.5: - MRM (629.1 → 553.2)
13: isoliquiritigenin_pos; 13.8: + MRM (257.0 → 137.0)
14: glycyrrhizic acid_neg; 15.0: - MRM (821.4 → 351.0)
15: glabridin_pos; 19.5: + MRM (325.1 → 189.1)
16: glycyrrhetinic acid_pos; 20.9: + MRM (471.2 → 189.1)
Relative absorbance
Counts (%) vs. Acquisition Time (min)

C

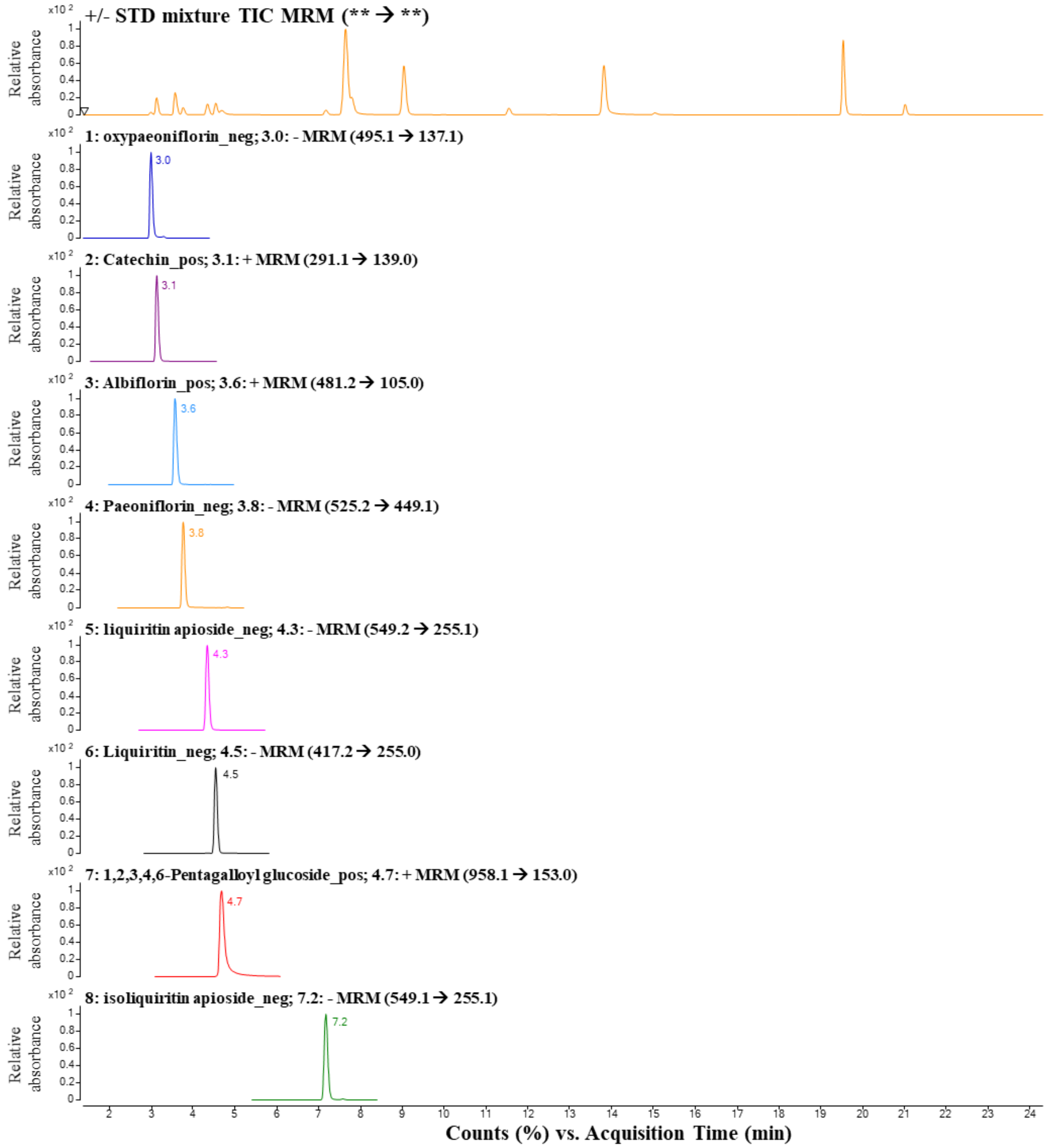

+/- STD mixture TIC MRM (** → **)
1: oxypaeoniflorin_neg; 3.0: - MRM (495.1 → 137.1)
2: Catechin_pos; 3.1: + MRM (291.1 → 139.0)
3: Albiflorin_pos; 3.6: + MRM (481.2 → 105.0)
4: Paeoniflorin_neg; 3.8: - MRM (525.2 → 449.1)
5: liquiritin apioside_neg; 4.3: - MRM (549.2 → 255.1)
6: Liquiritin_neg; 4.5: - MRM (417.2 → 255.0)
7: 1,2,3,4,6-Pentagalloyl glucoside_pos; 4.7: + MRM (958.1 → 153.0)
8: isoliquiritin apioside_neg; 7.2: - MRM (549.1 → 255.1)
Relative absorbance
x10 2
Counts (%) vs. Acquisition Time (min)

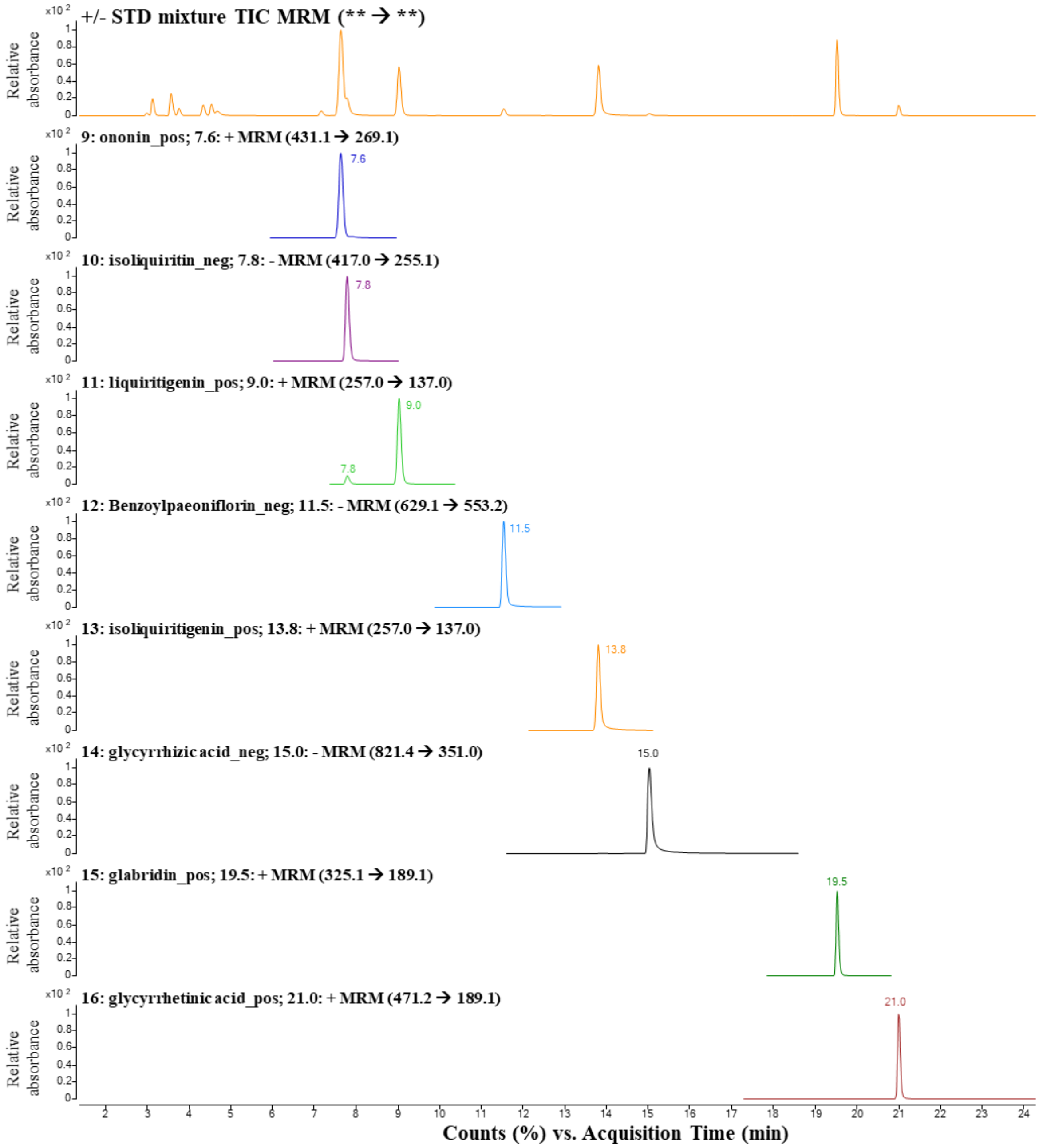

Figure S3. Dynamic multiple reaction monitoring (MRM) mode of JGT-W **(A)**, JGT-E **(B)**, and standard mixture **(C)** using an UHPLC-TQ-MS/MS.

Figure S4

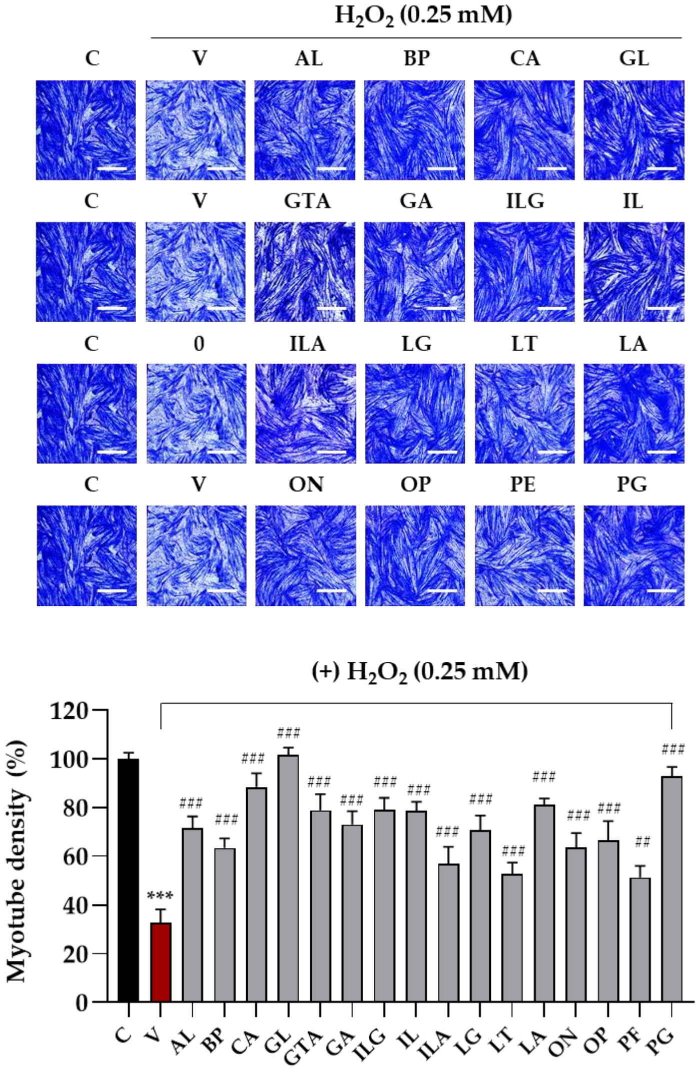

Figure S4. Muscle-protective activity of 16 compounds in JGT. C2C12 myotubes were pretreated with non-toxic concentrations of each compounds for 12 h, and then exposed to 0.25 mM H_2O_2 for an additional 40 h. After staining with crystal violet solution, myotube density was quantified. Data are presented as the mean ± SD (n = 3). Statistical significance was evaluated by one-way ANOVA followed by Dunnett's multiple comparison test. ***$p < 0.001$ *vs*. vehicle-treated controls; ##$p < 0.01$, ###$p < 0.001$ *vs*. H_2O_2 + vehicle-treated control cells. C, control; V, vehicle; AL, Albiflorin; BP, Benzoylpaeoniflorin; CA, Catechin; GL, Glabridin; GTA, Glycyrrhetinic acid; GA, Glycyrrhizic acid; ILG, Isoliquiritigenin; IL, Isoliquiritin; ILA, Isoliquiritin apioside; LG, Liquiritigenin; LT, Liquiritin; LA, Liquiritin apioside; ON, Ononin; OP, Oxypaeoniflorin; PF, Paeoniflorin; PG, 1,2,3,4,6-O-Pentagalloylglucose. Scale bar = 100 μm.

Table S1

Table S1. Recovery data of the 16 compounds in JGT (n = 3)

No.	Compound	Original amount (μg/mL)	Spiked amount (μg/mL)	Observed amount (μg/mL)	Recovery* (%)	CV (%)
**1**	Oxypaeoniflorin	0.490	0.313	0.805	101.0	0.01
			0.625	1.118	100.6	0.44
			1.25	1.709	97.6	2.16
**2**	Catechin	0.620	0.0313	0.913	93.8	0.02
			0.625	1.250	100.8	2.03
			1.25	1.828	96.6	0.69
**3**	Albiflorin	0.728	0.0313	1.049	102.7	0.06
			0.625	1.348	99.2	4.51
			1.25	1.944	97.3	1.29
**4**	Paeoniflorin	2.19	0.625	2.831	102.8	0.12
			1.25	3.495	104.5	2.06
			2.5	4.55	94.5	0.70
**5**	Liquiritin apioside	3.19	0.625	4.07	141.1	0.02
			1.25	4.43	99.2	0.14
			2.5	5.56	94.9	0.55
**6**	Liquiritin	2.45	0.625	3.26	131.3	0.03
			1.25	3.700	100.4	0.43
			2.5	4.748	92.1	0.53
**7**	1,2,3,4,6-Pentagalloyl glucoside	1.235	0.625	1.878	102.8	0.02
			1.25	2.544	104.7	1.33
			2.5	3.702	98.6	2.04
**8**	Isoliquiritin apioside	3.34	0.625	4.207	138.8	0.01
			1.25	4.631	103.2	0.55
			2.5	5.664	93.0	0.68
**9**	Ononin	0.703	0.313	1.022	102.1	0.01
			0.625	1.373	107.2	1.30
			1.25	1.955	100.2	0.62
**10**	Isoliquiritin	1.09	0.313	1.426	107.8	0.01
			0.625	1.820	108.9	0.40
			1.25	2.285	95.7	0.72
**11**	Liquiritigenin	0.195	0.313	0.507	99.9	0.01
			0.625	0.831	101.7	0.63
			1.25	1.425	98.4	0.29
**12**	Benzoylpaeoniflorin	0.626	0.313	0.941	100.8	0.01
			0.625	1.267	102.6	1.87
			1.25	1.873	99.8	0.97
**13**	Isoliquiritigenin	0.206	0.313	0.509	97.1	0.004
			0.625	0.839	101.2	2.46
			1.25	1.465	100.7	0.53
**14**	Glycyrrhizic acid	2.59	0.625	3.242	103.9	0.08
			1.25	3.832	99.1	0.7
			2.5	5.256	106.5	8.12
**15**	Glabridin	0.444	0.313	0.903	100.0	0.03
			0.625	1.103	105.3	2.80

			1.25	1.815	101.3	1.39
**16**	Glycyrrhetinic acid	0.068	0.313	0.404	107.4	0.01
			0.625	0.688	99.1	1.07
			1.25	1.362	103.5	1.83

*Recovery (%) = {(Observed amount - Original amount)/spiked amount} × 100
